# Is occupational physical activity associated with mortality in UK Biobank?

**DOI:** 10.1186/s12966-021-01154-3

**Published:** 2021-07-27

**Authors:** Matthew Pearce, Tessa Strain, Katrien Wijndaele, Stephen J. Sharp, Alexander Mok, Søren Brage

**Affiliations:** MRC Epidemiology Unit, University of Cambridge School of Clinical Medicine, Level 3 Institute of Metabolic Science, Addenbrooke’s Treatment Centre, Cambridge Biomedical Campus, Cambridge, CB2 0SL UK

**Keywords:** Paradox, Labour, Heavy, Leisure-time

## Abstract

**Background:**

Current physical activity guidelines do not distinguish between activity accumulated in different behavioural domains but some studies suggest that occupational physical activity (OPA) may not confer health benefits and could even be detrimental. The purpose of this study was to investigate associations between OPA and mortality outcomes.

**Methods:**

From baseline (2006–2010), 460,901 UK Biobank participants (aged 40–69 years) were followed for a median 12.0 (IQR: 11.3–12.7) years. OPA was categorised by cross-tabulating degree of manual work and walking/standing work amongst those in paid employment (*n* = 267,765), and combined with categories of occupational status for those not in paid employment (*n* = 193,136). Cox proportional hazards models were used to estimate sex-stratified hazard ratios (HR) and 95% confidence intervals (CI) for mortality from all causes, CVD, and cancer by occupational group, and for working hours/week and non-occupational physical activity stratified by occupational group. Models included adjustment for age and a range of lifestyle, socio-economic and health-related covariates.

**Results:**

During 5,449,989 person-years of follow-up, 28,740 deaths occurred. Compared to those reporting no heavy manual or walking/standing work (e.g. sedentary office workers) and adjusting for covariates, retirement was associated with lower mortality in women (HR = 0.62, CI: 0.53–0.72) and men (HR = 0.80, CI: 0.71–0.90), whereas unemployment was associated with higher mortality in men only (HR = 1.24, CI: 1.07–1.45). Within the working population, there was no evidence of differences in all-cause, CVD or cancer mortality by OPA group when comparing those reporting higher levels of OPA to the lowest OPA reference group for both women and men. Working < 35 h/week versus 35–40 h/week was associated with lower mortality in women (HR = 0.85, CI: 0.79–0.92) and men (HR = 0.83, CI: 0.78–0.89), with no interaction by OPA. Non-occupational physical activity was associated with lower mortality in working women (HR = 0.89 per 5 kJ/day/kg, CI: 0.84–0.95) and men (HR = 0.87 per 5 kJ/day/kg, CI: 0.84–0.91), with no interaction by OPA group.

**Conclusions:**

Jobs classified as higher levels of OPA may not be as active as reported, or the types of physical activity performed in those jobs are not health-enhancing. Irrespective of OPA category or employment status, non-occupational physical activity appears to provide health benefits.

**Supplementary Information:**

The online version contains supplementary material available at 10.1186/s12966-021-01154-3.

## Background

The benefits of physical activity are well established [1, 2], with guidelines from the UK Chief Medical Officers [3] and the World Health Organization [4] (WHO) recommending the equivalent of 150 min of moderate intensity or 75 min of vigorous intensity physical activity each week for maintenance of good physical and mental health. No distinctions are made between physical activity in leisure-time, transport, home, education, or occupational domains; the total volume of activity is assumed to be beneficial regardless of the domain in which it is accrued. Contradictory to this advice is the suggestion that occupational physical activity (OPA) does not confer the same benefits, and may even be harmful to health [5]. One meta-analysis reported that male (but not female) workers with high level OPA were at 18% higher risk of all-cause mortality compared to those at low levels [6]. Proposed reasons for these findings include OPA being performed at lower intensities, for protracted periods, and in a static posture [7], although the existing evidence also has weaknesses including the use of crude self-reported OPA measures, lack of adjustment for non-occupational physical activity, residual confounding for socio-economic status and lifestyle factors (e.g. smoking), and limited geographical representation [8, 9]. Prior studies within occupational strata have reported that active jobs were associated with lower mortality [10, 11].

UK Biobank is a large prospective cohort study incorporating assessment of total and domain-specific physical activity data as well as occupational variables. Together, these variables allow researchers to estimate OPA exposure in different ways and conduct complimentary sets of analyses to examine associations between OPA and health outcomes. Domain-specific physical activity variables can be combined to categorise the OPA type (e.g. walking, standing, manual) of those in paid employment and examine differences in mortality outcomes between groups. The importance of associations between OPA and mortality can be viewed in a broader context by including groups who are unexposed to OPA for different reasons (e.g. retired, unemployed, in education). Duration of work per week is another dimension which provides an estimate of the dose of OPA. Studying how associations between dose and mortality vary across OPA groups may provide further insight and could better address issues of confounding patterned by occupation. Given the aforementioned sex differences of associations between OPA and mortality, analyses should also ideally be sex-stratified, and UK Biobank has sufficient sample size and accrued deaths to facilitate these analyses. A range of lifestyle, socio-economic and health-related variables are collected using a standardised protocol, and it is also possible to calibrate self-reported non-occupational physical activity to objective measures of physical activity using the accelerometer sub-cohort [12] to better control for physical activity outside of work. The strengths of UK Biobank offer multiple opportunities for improving our understanding of the role OPA and its relationship to health outcomes, particularly in the UK, where there are few contemporary analyses. The main aims of this study were to investigate: 1) differences in mortality outcomes by OPA groups; 2) heterogeneity of associations between dose of work in hours per week and mortality outcomes across OPA groups; 3) heterogeneity of associations between non-occupational physical activity and mortality outcomes across OPA group and work duration group.

## Methods

### Participants and study design

UK Biobank is an ongoing prospective cohort study of men and women aged 40–69 years residing within 25 miles of one of 22 assessment centres in England, Scotland, and Wales. Participants were identified from National Health Service (NHS) general practitioner registries and invited to a baseline assessment between 2006 and 2010 [13]. The study was approved by the North West Multicentre Research Ethics Committee and participants provided written informed consent. Data for the current analyses were updated on 20th April 2021, containing information from 502,488 participants with baseline measures. Participant exclusions are outlined in Supplementary Figure 1.

### Exposures

Occupational status, standard occupational classification (SOC) [14], degree of manual work, degree of standing/walking work, and work duration in hours per week were self-reported using a touch-screen questionnaire [15]. Participants with missing data for these exposure variables were excluded from analyses (*n* = 9362), as were those reporting paid employment status but zero working hours (*n* = 186).

#### Categorical OPA

For those in paid employment, degree of manual work and degree of standing/walking work were both reported as one of four categories: “never/rarely”, “sometimes”, “usually”, “always”. Responses of “usually” and “always” were collapsed for both manual work and standing/walking, with the two variables cross-tabulated (Supplementary Table 1) to create six mutually exclusive OPA groups: “no manual, no standing/walking”, “no manual, some standing/walking”, “no manual, usually standing/walking”, “some manual, some standing/walking”, “some manual, usually standing/walking”, “usually manual, usually standing/walking” [16]. To complete the categorisation of OPA group to also include those unexposed to work at baseline, we expanded the variable using the occupational status of those not in paid employment (retired, unable to work due to illness/disability, caring for home/family, student, unemployed, unpaid work). In preliminary testing, we examined the face validity of the six OPA groups in paid employment by comparing total physical activity estimated by wrist acceleration across the categories within the accelerometery sub-cohort, observing a trend of higher total physical activity by presumed higher OPA level (Supplementary Figure 2).

In supplementary analyses, we used an alternative categorisation using nine groups based on SOC code of those in paid employment (Supplementary Table 2), while including the same occupational status categories for those not in paid employment (retired, unemployed, etc.).

#### Work duration

Work duration in hours per week was recorded only by those in paid employment and used to estimate the dose of OPA. Fractional polynomials showed that work duration did not meet the log-linear assumption so this variable was categorised using tertiles (< 35, 35–40, > 40 h per week).

#### Non-occupational physical activity

We have previously shown how self-reported variables representing multiple behaviours can be combined to predict total PAEE in UK Biobank [12]. For the present study, values were set to zero for the three occupational activity variables (durations of heavy manual or physical work, standing/walking work, and sedentary work) included in this prediction model such that the resulting estimate represents non-occupational PAEE rather than total PAEE. Further details of this prediction model are shown in Supplementary Table 3 [17].

### Outcome assessment

Vital status, date of death, and cause of death were established by linkage to national death registries obtained from the Health and Social Care Information Centre for England and Wales and the Information Services Department for Scotland [13]. The censoring date for mortality was 28th February 2021 in all three nations. International Classification of Diseases 10th edition (ICD-10) codes were used to attribute causes of death from cardiovascular disease (CVD, ICD-10 I00–99) and cancer (ICD-10 C00–97).

### Covariate assessment

Demographic, lifestyle, and clinical variables were assessed at baseline by the aforementioned touch-screen questionnaire, verbal interview, or physical measurement. The following baseline variables were considered as potential confounders of the association between OPA and mortality: age (years), sex, ethnic background (white, not-white, prefer not to answer, do not know), Townsend deprivation index (higher scores indicating higher levels of deprivation), highest educational level (degree or above, any other qualification, no qualification, prefer not to answer), annual household income (<£18,000, £18,000–£30,999, £31,000–£51,999, £52,000–£100,000, >£100,000, prefer not to answer, do not know), work duration (when not the main exposure or stratification variable; tertiles of < 35, 35–40, > 40 h per week), time in current job (years), job involves shift work (never, sometimes, usually/always, prefer not to answer, do not know), alcohol consumption (never, previous, current, prefer not to answer), smoking (never, previous, current, prefer not to answer), adds salt to food (never, sometimes, usually/always, prefer not to answer), oily fish intake (never, sometimes, usually/always, prefer not to answer, do not know), fruit and vegetable intake (score from 0 to 4 with 1 point for ≥2 servings/day for each of fresh fruit, dried fruit, cooked vegetable, raw vegetable), processed and red meat intake (average weekly frequency in days per week), parental history of cancer or CVD (derived by collapsing 4 questions on cancer and CVD in mother and father; yes/no), use of blood pressure or cholesterol lowering medications (yes, no, prefer not to answer), doctor-diagnosed diabetes or treatment with insulin (yes, no, prefer not to answer), baseline CVD (self-reported or ICD-10 code I00–99 recorded in NHS Hospital Episode Statistics; yes/no), baseline cancer (self-reported or ICD-10 code C00–97 recorded in NHS Hospital Episode Statistics; yes/no), non-occupational PAEE (when not the main exposure; kJ/day/kg), body mass index (BMI) calculated from measured height and weight and included in models in three categories (< 25, 25–30, > 30 kg•m^− 2^), and resting heart rate measured in the sitting position using a blood pressure monitor (Omron 705 IT, OMRON Healthcare Europe B. V, Hoofddorp, Netherlands). Mean of two resting heart rate values was used except when only one value was available, in which case this was used.

### Statistical analyses

We used Cox proportional hazards model with age as the underlying timescale to estimate hazard ratios (HR) and 95% confidence intervals (CI) for the associations between the exposures and mortality outcomes. Two sets of covariate adjustments were used: Model 1 included all covariates listed above except BMI and resting heart rate which may be on the causal pathway between physical activity and mortality, while Model 2 included additional adjustment for BMI and resting heart rate.

For the first aim, we investigated the sex-stratified associations between OPA group and all-cause mortality (Model 1 and 2), CVD mortality (Model 2 only), and cancer mortality (Model 2 only). We repeated the all-cause mortality analysis excluding those with prevalent CVD or cancer at baseline (*n* = 84,974 excluded) and excluding those with time in current job less than 10 years (*n* = 71,042 excluded). In supplementary analyses, we also repeated the all-cause mortality analysis using an alternative grouping based on SOC codes. These analyses included the same groupings for those not in paid employment, as described above.

For the second aim, we investigated the sex-stratified associations between tertile of work duration and mortality outcomes within each OPA group (Models 1 and 2 for all-cause mortality, Model 2 for CVD and cancer mortality). In supplementary analyses, we also repeated the all-cause mortality analysis using an alternative grouping based on SOC codes. Groups not in paid employment were not included in any of these analyses.

For the third aim, we investigated the sex-stratified associations between non-occupational PAEE and mortality outcomes within each OPA group (Models 1 and 2 for all-cause mortality, Model 2 for CVD and cancer mortality). These analyses included groups not in paid employment. We then repeated the all-cause mortality analysis but instead of stratifying by OPA group, we stratified by tertile of work duration per week, excluding those not in paid employment. Hazard ratios for non-occupational PAEE were presented per 5 kJ/day/kg increments; this difference in activity volume is equivalent to an additional 21 min per day or 150 min of moderate intensity activity per week [4].

The proportional hazards assumption for categorical covariates was examined using log-log plots; the baseline hazard function was stratified by levels of those variables that did not satisfy this assumption (fruit and vegetable intake, income, education), while meat consumption (log [x + 1]) and Townsend index ([x]^2^) were transformed following inspection of fractional polynomials. We assessed the linearity of associations between non-occupational physical activity and all-cause mortality by adding a quadratic term to the Cox proportional hazards model. We then plotted the dose-response association for working and non-working women and men, as well as any smaller subgroup for which the quadratic term was statistically significant (*p* < 0.05, see Supplementary Figure 3). Non-occupational physical activity was modelled linearly as an approximation of these associations and to facilitate comparisons between strata. Variance inflation factors and Pearson correlations indicated no strong collinearity between variables. All analyses were stratified by sex *a priori*, based on findings from previous studies [6]. Wald tests were used to examine the potential interactions of work duration and non-occupational physical activity by strata. Individuals with missing covariate data (*n* = 29,873) were excluded, as were those who died in the first 2 years of follow-up (*n* = 2166) to mitigate potential reverse causation. All analyses were performed using STATA/SE 16.1 (StataCorp, TX, USA).

## Results

Baseline characteristics of the 460,901 included participants by sex and occupational status are shown in Table 1, and further details for OPA groups are provided in Supplementary Tables 4, 5, 6 and 7. Approximately one third of the participants were retired. Those in paid employment were younger, reported lower frequency of medication usage and had a lower prevalence of diabetes, CVD, or cancer at baseline. There were only minor differences between those in/not in paid employment with respect to resting heart rate, BMI, and lifestyle variables. Jobs involving no manual work or standing/walking were most common (women 38%; men 34%), whereas jobs involving the highest levels of manual work and standing/walking were less common (women 9%; men 16%). Supplementary Table 8 shows the distribution of participants across OPA groups within SOC code strata. Participants in managerial, professional, and administrative SOC codes tended to report less manual work and standing/walking, whereas participants in elementary, skilled trade, personal service, and operative SOC codes tended to report more manual work.

**Table 1:** Baseline characteristics of women and men in UK Biobank

	No paid employment		In paid employment		All	
	Women	Men	Women	Men	Women	Men
n (%)	109,591 (58)	78,869 (42)	138,062 (52)	126,362 (48)	247,653 (55)	205,231 (45)
Working hours per week, median (IQR)	0 (0;0)	0 (0;0)	35 (22;40)	40 (37;45)	15 (0;36)	15 (0;36)
Age in years at baseline, mean (SD)	61 (6)	62 (6)	52 (7)	53 (7)	56 (8)	57 (8)
White ethnicity, %	96	96	95	95	95	95
*Highest educational level*						
No qualification, %	26	26	8	10	16	16
Any other qualification, %	50	45	53	51	52	49
Degree or above, %	24	29	38	39	32	35
Townsend index (higher for more deprived), median (IQR)	−2.4 (−3.8;0.1)	−2.3 (−3.7;0.5)	− 2.1 (−3.6;0.4)	−2.2 (−3.7;0.3)	−2.2 (−3.7;0.3)	−2.2 (−3.7;0.4)
Household income before tax*						
Prefer not to answer, %	14	9	8	6	10	7
Do not know, %	9	3	3	1	5	2
< £18,000, %	31	35	11	7	20	18
£18,000–£30,999, %	24	27	21	19	22	22
£31,000–£51,999, %	13	17	28	30	22	25
£52,000–£100,000, %	6	7	24	30	16	21
> £100,000, %	2	1	6	8	4	6
*Occupational category*						
Retired, %	80	82			35	32
Caring for home/family, %	11	1			5	1
Unable to work due to illness, %	5	9			2	4
Unemployed, %	2	6			1	2
Unpaid work, %	1	1			1	0
Student, %	1	0			0	0
No manual, no standing/walking, %			38	34	21	21
No manual, Some standing/walking, %			23	22	13	14
No manual, Mostly standing/walking, %			12	7	7	4
Some manual, some standing/walking, %			6	9	4	5
Some manual, mostly standing/walking, %			12	12	7	7
Mostly manual, mostly standing/walking, %			9	16	5	10
*Smoking status*						
Never, %	58	42	61	54	60	50
Previous, %	34	46	30	34	32	39
Current, %	8	12	9	12	9	12
*Alcohol use status*						
Never, %	7	3	4	2	5	2
Previous, %	4	4	3	3	3	3
Current, %	89	93	93	95	91	94
Fruit/vegetable score, median (IQR)	2 (1;3)	1 (1;2)	2 (1;3)	1 (0;2)	2 (1;3)	1 (1;2)
Red/processed meat score, median (IQR)	1 (1;1)	1 (1;1)	1 (1;1)	1 (1;1)	1 (1;1)	1 (1;1)
Adds salt to food, %	38	41	38	41	38	41
Consumes oily fish, %	28	31	35	38	32	35
Non-occupational PAEE (kJ/day/kg), mean (SD)	43 (3)	42 (3)	44 (3)	43 (4)	44 (3)	42 (4)
Parental history of CVD or cancer, %	76	72	69	66	72	68
Blood pressure or cholesterol medication, %	33	47	14	23	23	33
Diagnosis of diabetes or insulin prescription, %	5	10	2	5	4	7
Prevalent CVD or cancer at baseline, %	18	24	9	10	13	15
*Body mass index*						
< 25 kg/m2, %	36	25	43	26	40	25
25–30 kg/m2, %	39	49	35	50	37	50
> 30 kg/m2, %	25	26	22	25	23	25
Resting heart rate in bpm, mean (SD)	71 (11)	69 (12)	70 (10)	68 (11)	70 (11)	68 (12)

*bpm* beats per minute, *CVD* cardiovascular disease, *IQR* interquartile range, *OPA* occupational physical activity, *PAEE* physical activity energy expenditure, *SD* standard deviation

*Values for other categorical variables using original UK Biobank coding are much smaller, so these are not reported in the table. Values for responses of “Do not know” / “Prefer not to answer” in other categorical variables: ethnic background (*n* = 1180 / 131), consumes oily fish (*n* = 1924 / 0), job involves shift work (*n* = 237 / 97), education (*n* = 0 / 4437), smoking status (*n* = 0 / 1433), alcohol use status (*n* = 0 / 364), adds salt to food (*n* = 0 / 229), blood pressure or cholesterol medication (*n* = 0 / 519), diagnosis of diabetes or insulin prescription (*n* = 0 / 265)

During a median 12.0 (IQR: 11.3–12.7) years of follow up (5,449,989 person-years), 28,740 deaths occurred. The majority of these deaths occurred in the participants not in paid employment, crude mortality rates being 673 per 100,000 person-years in retired women and 1215 per 100,000 person-years in retired men.

**Fig. 1:**
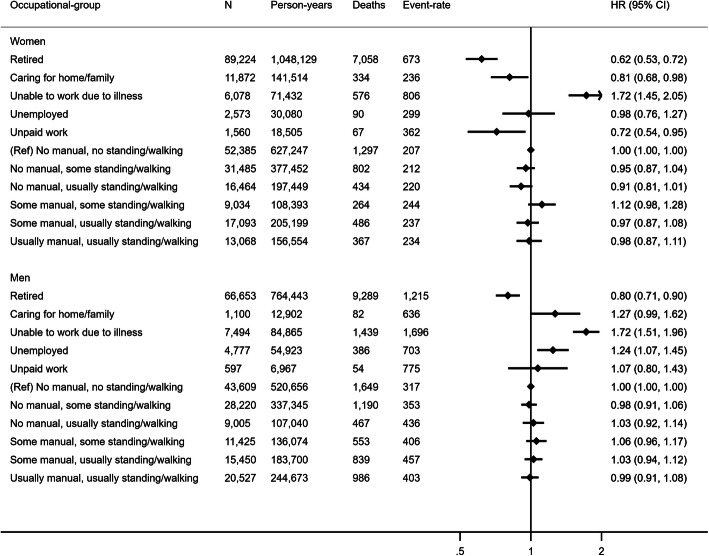
Hazard ratio (HR) and 95% confidence interval (CI) of all-cause mortality by occupational group. Reference group is “no manual, no standing/walking”. Model 1 hazard ratios are adjusted for age (underlying timescale), ethnicity, Townsend deprivation index, highest educational level (stratified baseline hazard), annual household income (stratified baseline hazard), working hours per week, years in current job, job involves shift work, alcohol consumption, smoking, salt added to food, oily fish intake, fruit and vegetable intake (stratified baseline hazard), processed and red meat intake, non-occupational physical activity energy expenditure, parental history of cancer or cardiovascular disease, use of blood pressure or cholesterol lowering medications, doctor-diagnosed diabetes or treatment with insulin, baseline prevalent cancer, baseline prevalent cardiovascular disease. Results for students not shown due to small numbers of events. Arrow indicates confidence interval boundary out of range. Event-rate per 100,000 person-years

Figure 1 shows adjusted hazard ratios and 95% confidence intervals of all-cause mortality for OPA groups compared with the referent group of being in a paid job involving no manual work or standing/walking (e.g. sedentary office work). Among women and men in paid employment, there were no differences in hazard of all-cause mortality across OPA groups. Compared to the referent group, the adjusted hazard of all-cause mortality was lower in retired women, but twice as high in women unable to work due to illness. The hazard was higher in men unable to work due to illness, unemployed men, and in men caring for home or others. Additional adjustment for resting heart rate and BMI did not alter these findings (Supplementary Figure 4). We observed similar results when excluding prevalent CVD or cancer at baseline (Supplementary Figure 5) and excluding those with time in current job less than 10 years (Supplementary Figure 6).

Among participants in paid employment, there were no differences in hazard of CVD or cancer mortality between OPA groups (Supplementary Figure 7). For those not in paid employment, hazards for CVD mortality were similar to those observed for all-cause mortality albeit with greater uncertainty: lower hazards in the retired and higher hazards in those unable to work due to illness. Hazard of cancer mortality showed a similar pattern to results for all-cause mortality except that confidence intervals of the hazard for retired men crossed zero.

When OPA groups were replaced with SOC code groups for those in paid employment (Supplementary Figure 8), men with “elementary” or “process, plant or machine operative” SOC codes had higher hazards of all-cause mortality than those in “senior managerial positions” (the category we assumed to be most similar to sedentary desk work with large numbers in both sexes), however no such associations were observed in women. Similar associations were observed in the model adjusting for resting heart rate and BMI (Supplementary Figure 8).

**Fig. 2:**
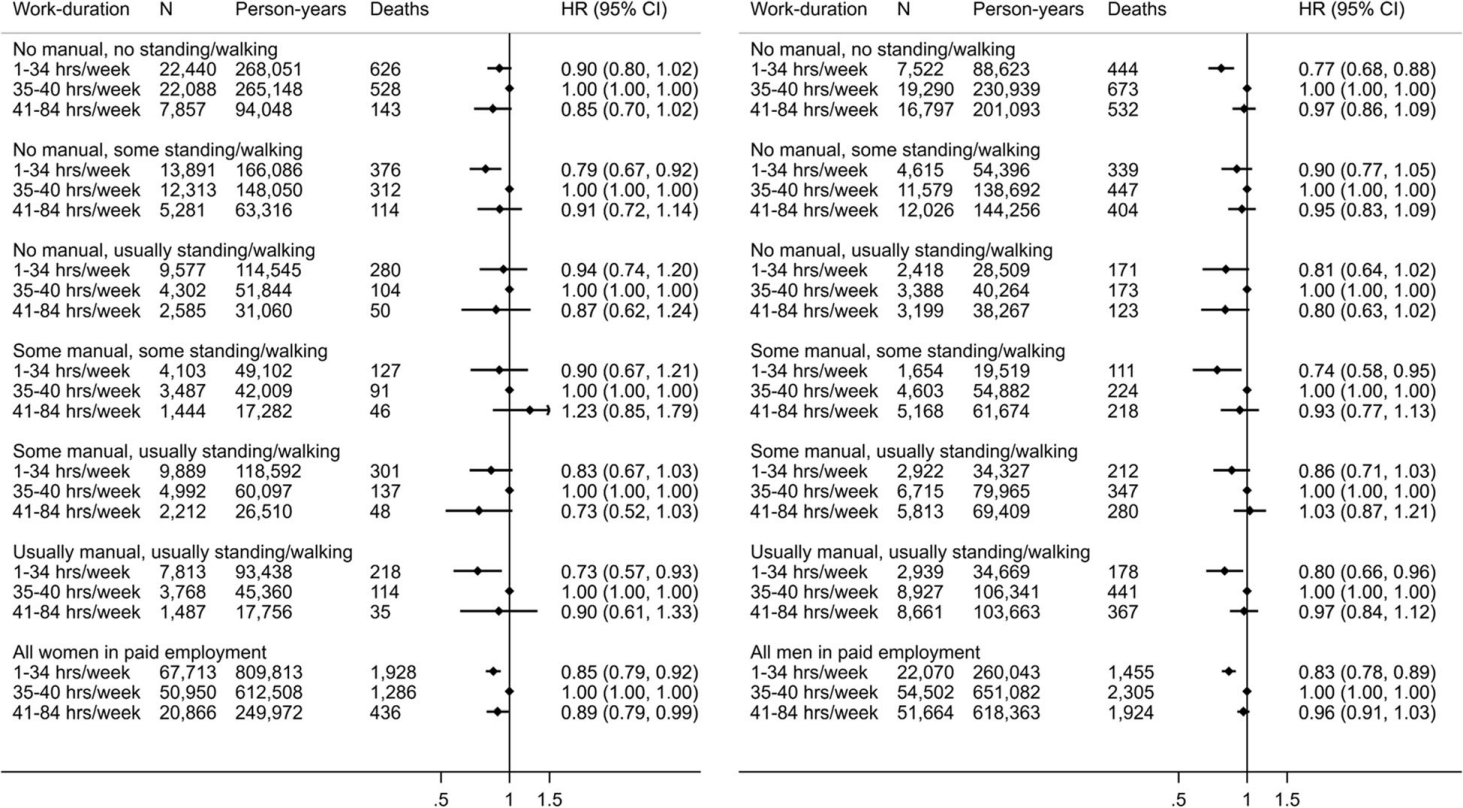
Hazard ratio (HR) and 95% confidence interval (CI) of all-cause mortality by tertile of work duration in hours per week across occupational physical activity strata in women (left) and men (right) in paid employment. Reference group is “35-40 hours per week”. Model 1 hazard ratios are adjusted for age (underlying timescale), ethnicity, Townsend deprivation index, highest educational level (stratified baseline hazard), annual household income (stratified baseline hazard), years in current job, job involves shift work, alcohol consumption, smoking, salt added to food, oily fish intake, fruit and vegetable intake (stratified baseline hazard), processed and red meat intake, non-occupational physical activity energy expenditure, parental history of cancer or cardiovascular disease, use of blood pressure or cholesterol lowering medications, doctor-diagnosed diabetes or treatment with insulin, baseline prevalent cancer, baseline prevalent cardiovascular disease. Arrow indicates confidence interval boundary out of range

Figure 2 shows hazards of all-cause mortality for tertiles of weekly work duration, stratified by OPA group. Women working 1–34 h per week had lower hazard than those working 35–40 h per week, and women working the longest hours also had lower hazard than those in the middle tertile. Among men, working 1–34 h per week was associated with lower hazards of all-cause mortality, compared with those working 35–40 h per week but there was no difference between those working 35–40 vs > 40 h per week. There was no evidence of interaction between work duration and OPA group (*p* = 0.75 and *p* = 0.59 for women and men, respectively). Additional adjustment for resting heart rate and BMI did not materially alter these findings (Supplementary Figure 9). For CVD and cancer mortality in both women (Supplementary Figure 10) and men (Supplementary Figure 11), working 1–34 h per week was associated with lower hazards compared with those working 35–40 h per week, but there was no difference between those working 35–40 vs > 40 h per week. There was no evidence of interaction between weekly work duration and SOC code group (*p* = 0.14 and *p* = 0.76 for women and men, respectively; Supplementary Figure 12).

**Fig. 3:**
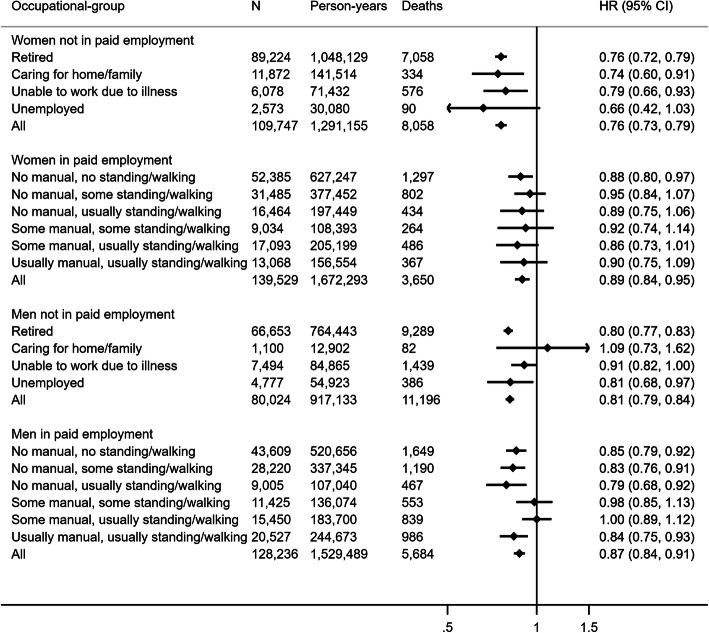
Hazard ratio (HR) and 95% confidence interval (CI) of all-cause mortality per 5 kJ/day/kg of non-occupational physical activity energy expenditure across occupational strata. Model 1 hazard ratios are adjusted for age (underlying timescale), ethnicity, Townsend deprivation index, highest educational level (stratified baseline hazard), annual household income (stratified baseline hazard), working hours per week, years in current job, job involves shift work, alcohol consumption, smoking, salt added to food, oily fish intake, fruit and vegetable intake (stratified baseline hazard), processed and red meat intake, parental history of cancer or cardiovascular disease, use of blood pressure or cholesterol lowering medications, doctor-diagnosed diabetes or treatment with insulin, baseline prevalent cancer, baseline prevalent cardiovascular disease. Results for students and unpaid workers not shown due to small numbers of events. Arrow indicates confidence interval boundary out of range

Figure 3 shows associations between non-occupational PAEE and all-cause mortality across occupational strata. For participants in paid employment, non-occupational PAEE was associated with lower hazard of all-cause mortality in both sexes with no evidence of interaction by OPA group (*p* = 0.66 and *p* = 0.12 for women and men, respectively). For participants not in paid employment, non-occupational PAEE (i.e., their total PAEE) was also associated with lower hazard of all-cause mortality in both sexes with evidence of interaction by occupational status in women (*p* = 0.04) but not men (*p* = 0.14). Following additional adjustment for resting heart rate and BMI, hazard ratios were attenuated across all strata (Supplementary Figure 13). Non-occupational PAEE was associated with lower hazard of CVD mortality in women and men both in/not in paid employment. Similar results were observed for cancer mortality except that there was no association between non-occupational PAEE and cancer mortality amongst women in paid employment (Supplementary Figure 14).

**Fig. 4:**
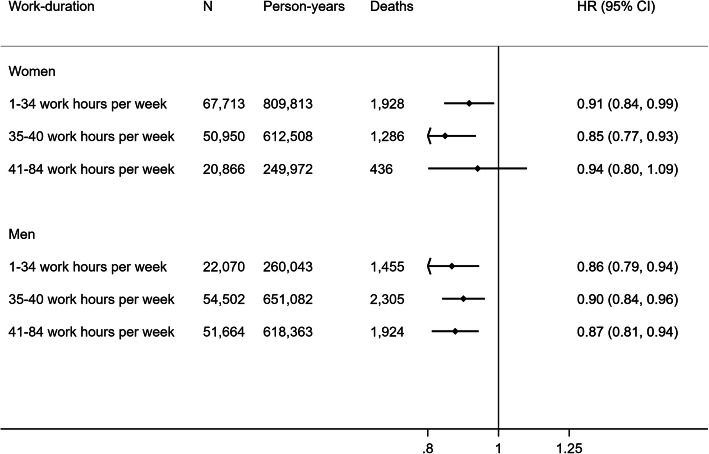
Hazard ratio (HR) and 95% confidence interval (CI) of all-cause mortality per 5 kJ/day/kg of non-occupational physical activity energy expenditure across tertiles of work duration in hours per week. Model 1 hazard ratios are adjusted for age (underlying timescale), ethnicity, Townsend deprivation index, highest educational level (stratified baseline hazard), annual household income (stratified baseline hazard), years in current job, job involves shift work, alcohol consumption, smoking, salt added to food, oily fish intake, fruit and vegetable intake (stratified baseline hazard), processed and red meat intake, parental history of cancer or cardiovascular disease, use of blood pressure or cholesterol lowering medications, doctor-diagnosed diabetes or treatment with insulin, baseline prevalent cancer, baseline prevalent cardiovascular disease, occupational physical activity category. Arrow indicates confidence interval boundary out of range

Figure 4 shows the inverse association between non-occupational PAEE and all-cause mortality which was reasonably consistent across tertiles of weekly work duration with no evidence of interaction in either women (*p* = 0.33) or men (*p* = 0.65) (Fig. 4). Following additional adjustment for resting heart rate and BMI, hazard ratios were attenuated across all strata (Supplementary Figure 15). Results for CVD and cancer mortality were similar but with wider confidence intervals and the same lack of an association observed amongst women in paid employment (Supplementary Figure 16).

## Discussion

In this study of 460,901 women and men including 267,765 paid workers in occupations with varying degrees of manual work and standing/walking, we found little evidence that all-cause mortality, cancer mortality, or CVD mortality varied by OPA group in either sex. Working part-time rather than full-time was associated with lower hazard of mortality but there was no evidence indicating that longer weekly work duration was more harmful in some OPA groups than others. Retirement was associated with lower mortality in both men and women but being unable to work due to illness at baseline was predictably not beneficial for survival. Non-occupational physical activity was beneficial for women and men in paid employment with no interaction with OPA group, supporting universal physical activity guidelines [3].

We found no evidence of an association between OPA and all-cause mortality, CVD mortality, or cancer mortality in either sex after controlling for non-occupational physical activity, weekly work duration, and a range of demographic, clinical, and lifestyle variables. This is somewhat in contrast to a meta-analysis of 193,696 people reporting that men with high level of OPA were at higher risk of all-cause mortality than those at the low level (HR = 1.18, 95% CI: 1.05–1.34, I^2^ = 76%), and the corresponding result for women which showed some evidence of an inverse association (HR = 0.90, 95% CI: 0.80–1.01, I^2^ = 0%) [6]. Supplementary analyses in a subsample of included studies from the same report showed no association in either sex when a high level of OPA was compared to sedentary rather than low OPA reference group. Our main findings from a single but larger UK cohort using a sedentary OPA reference group are more aligned with this second set of results as well as other studies from Europe [18–22] and the USA [23] indicating no association, but contrast with findings from the Copenhagen General Population Study showing greater all-cause mortality risk with higher OPA, and the longer survival time of Norwegian men (but not women) in occupations characterised by walking/lifting and heavy labour. Discrepancies between our findings and previous work, as well as inconsistent findings in the published literature, may partly be explained by variation in working patterns and conditions between populations and eras. For example, the strongest effect size (HR 3.40, 95% CI: 1.94–5.96) in the above meta-analysis is from a Taiwanese study with baseline in 1990 [24], likely not generalisable to the UK between 2006 and 2010. Alternatively, our findings perhaps indicate that in this UK population, the combination of two self-reported variables is insufficient to characterise the physical activity profile of work throughout the day or week, making groups more difficult to distinguish and biasing effect estimates towards the null. In our SOC code analysis, we did observe higher all-cause mortality for “elementary occupations” and “process, plant and machine operatives” in men. These jobs (including assembly line and construction workers, cleaners, and drivers) are perhaps more consistent in terms of activity type and intensity and thus better characterised. The potential risks of the physical activity performed as part of these occupations should be investigated further using objective measures of physical activity labelled by domain. Accelerometers have been combined with work diaries to show that for mostly (71%) “blue-collar” workers in Denmark, reallocating time to moderate-to-vigorous activity (MVPA) at work was positively associated with long -term sickness absence, whereas an inverse association was observed for reallocating time to MVPA in leisure-time [25].

In contrast to objective monitoring, self-reported categorical data do not detail the pattern of work bouts or intensity across each day. Although a strength of this work was calibration of our estimate of non-occupational PAEE to objective measures, we have previously shown that the combined inference of activity volume from these self-report data is weak relative to objective measures, with a large proportion of unexplained variance typical of self-report data [26]. This unexplained variance would include unmeasured OPA which could vary by occupation. Our method of classifying OPA by cross-tabulating and combining categories could also mask associations in smaller groups which tended to be engaged in more manual work. Without methods to more accurately estimate the dose of OPA and control for non-occupational activity, it is at present difficult to rule out the possibility of health risks or benefits associated with OPA, let alone make domain-specific health recommendations such as those relating to total physical activity, for which robust measures are available [27].

Overall, we found that non-occupational physical activity was inversely associated with all-cause mortality in paid workers, with stronger associations for those not in paid employment. We observed no interaction between OPA category and non-occupational PAEE in paid workers, reflecting previous reports that leisure-time physical activity was beneficial independent of occupational physical activity level [28, 29]. Taken together, our results for OPA and non-occupational physical activity suggest that all adults should aim to be active during their leisure-time irrespective of their occupational status, with the potential additional benefit of substituting out harmful sedentary behaviours [30]. Our results also indicate that OPA may not confer health benefits in this relatively older UK population so the message to be active in leisure-time may be even more important. Moreover, increasing activity at work may be difficult for some workers.

Strengths of this work include a large single cohort study allowing robust estimation of associations, adjustment for a wide range of socio-economic and behavioural covariates as well as sufficient size to conduct stratified analyses larger than many occupational cohort studies. There are also important limitations of this work. As in any observational study, the above adjustments cannot fully eliminate confounding. Job satisfaction, exposure to hazardous materials or working conditions, and work shift pattern data are available in UK Biobank but only in a subsample. Data for phenomena which may potentially confound associations, such as work stress [31, 32] and access to sick leave [33] are not recorded. Characteristics like these may be patterned by occupational group, and these strata could be used to investigate specific working cohorts, such as in previous studies [10, 11]. The direct validity of the SOC code grouping used here is unknown and the high level categories used contain heterogeneous occupations (see Supplementary Table S2 for more details).

We used baseline data to assign occupational status and were unable to account for any changes during the follow-up period. In a sample aged 40–69 at baseline, retirement or changes in work due to illness during follow-up are of particular concern. Exposure to OPA may be determined by health status (healthy worker effect), for example selection of healthy workers into and remaining in more physically demanding jobs or unhealthy workers into less demanding jobs. Future work should therefore examine associations between change in OPA over time and mortality while accounting for changes in health status. We were also unable to account for potentially complex work histories leading up to baseline, account for changes to nature of work over time [34], or generalise our findings to younger workers. There is also evidence of a healthy volunteer selection bias in UK Biobank such that it is not representative of the general population [35], particularly in relation to smoking and education [36] which are notable confounders for this study.

## Conclusions

In summary, analysis of this population of UK adults aged 40–69 years old showed limited evidence of an association between OPA and mortality outcomes, although potential measurement error and residual confounding mean that we are unable to rule out the possibility of either health benefits or risks. Until stronger evidence is available from a combination of domain labels and objective assessment of the temporal pattern of activity, individuals should continue to maximise their physical activity volume during leisure-time irrespective of their occupation.

## Supplementary Information

**Additional file 1: Table S1.** Creation of joint standing/walking work and manual work variable. **Table S2.** Standard occupational classifications (SOC) codes of paid workers in UK Biobank. **Table S3.** Mutually adjusted sex-specific coefficients (standard errors) for prediction of average daily wrist acceleration (ln milli-g) from 13 self-reported behaviours. **Table S4.** Baseline characteristics of women in paid employment in UK Biobank. **Table S5.** Baseline characteristics of men in paid employment in UK Biobank. **Table S6.** Baseline characteristics of women not in paid employment in UK Biobank. **Table S7.** Baseline characteristics of men not in paid employment in UK Biobank. **Table S8.** Distribution of participants across occupational physical activity categories within strata of standard occupational code in women (*n*=139,529) and men (*n*=128,236) in UK Biobank. **Figure S1.** Flowchart detailing participant exclusions. **Figure S2.** Median, interquartile range, upper and lower adjacent values of average wrist acceleration in milli-g by occupational physical activity strata. **Figure S3.** Hazard ratio and 95% confidence intervals for association between non-occupational physical activity energy expenditure (PAEE) and all-cause mortality. **Figure S4.** Hazard ratio (HR) and 95% confidence interval (CI) of all-cause mortality by occupational group (Model 2). **Figure S5.** Hazard ratio (HR) and 95% confidence interval (CI) of all-cause mortality by occupational category excluding those with prevalent cardiovascular disease or cancer at baseline. **Figure S6.** Hazard ratio (HR) and 95% confidence interval (CI) of all-cause mortality by occupational category excluding paid workers with time in current job less than 10 years. **Figure S7.** Hazard ratio (HR) and 95% confidence interval (CI) of cardiovascular disease mortality (left) and cancer mortality (right) by occupational group (Model 2). **Figure S8.** Hazard ratio (HR) and 95% confidence interval (CI) of all-cause mortality by occupational group using Standard Occupational Classification code. **Figure S9.** Hazard ratio (HR) and 95% confidence interval (CI) of all-cause mortality by tertile of work duration in hours per week across occupational physical activity strata in women (left) and men (right) in paid employment (Model 2). **Figure S10.** Hazard ratio (HR) and 95% confidence interval (CI) of cardiovascular disease mortality (left) and cancer mortality (right) by tertile of work duration in hours per week across occupational physical activity strata for women in paid employment (Model 2). **Figure S11.** Hazard ratio (HR) and 95% confidence interval (CI) of cardiovascular disease mortality (left) and cancer mortality (right) by tertile of work duration in hours per week across occupational physical activity strata for men in paid employment (Model 2). **Figure S12.** Hazard ratio (HR) and 95% confidence interval (CI) of all-cause mortality by tertile of work duration in hours per week across standard occupational code strata for women (top) and men (bottom) in paid employment (Model 2). **Figure S13.** Hazard ratio (HR) and 95% confidence interval (CI) of all-cause mortality per 5 kJ/day/kg of non-occupational physical activity energy expenditure across occupational strata (Model 2). **Figure S14.** Hazard ratio (HR) and 95% confidence interval (CI) of cardiovascular disease mortality (left) and cancer mortality (right) per 5 kJ/day/kg of non-occupational physical activity energy expenditure across occupational strata (Model 2). **Figure S15.** Hazard ratio (HR) and 95% confidence interval (CI) of all-cause mortality per 5 kJ/day/kg of non-occupational physical activity energy expenditure across tertiles of working hours per week (Model 2). **Figure S16.** Hazard ratio (HR) and 95% confidence interval (CI) of cardiovascular disease mortality (left) and cancer mortality (right) per 5 kJ/day/kg of non-occupational physical activity energy expenditure across tertiles of work duration in hours per week (Model 2).

## Data Availability

The UK Biobank data that support the findings of this study are available to all bona fide researchers for health related research that is in the public interest, https://www.ukbiobank.ac.uk/register-apply/. This work was conducted under UK Biobank application number 20684.

## Abbreviations

BMI: Body mass index
CI: Confidence interval
HR: Hazard ratio
ICD-10: International Classification of Diseases 10th edition
MVPA: Moderate-to-vigorous physical activity
NHS: National Health Service
OPA: Occupational physical activity
PAEE: Physical activity energy expenditure
SOC: Standard occupational classification
UK: United Kingdom
