# Supplementary material for: Is occupational physical activity associated with mortality in UK Biobank?
Source: Int J Behav Nutr Phys Act. 2021 Jul 27;18:102. doi: 10.1186/s12966-021-01154-3 (PMC8314512; doi:10.1186/s12966-021-01154-3)
Supplement: Supplementary file 1 — Additional file 1: Table S1. Creation of joint standing/walking work and manual work variable. Table S2. Standard occupational classifications (SOC) codes of paid workers in UK Biobank. Table S3. Mutually adjusted sex-specific coefficients (standard errors) for prediction of average daily wrist acceleration (ln milli-g) from 13 self-reported behaviours. Table S4. Baseline characteristics of women in paid employment in UK Biobank. Table S5. Baseline characteristics of men in paid employment in UK Biobank. Table S6. Baseline characteristics of women not in paid employment in UK Biobank. Table S7. Baseline characteristics of men not in paid employment in UK Biobank. Table S8. Distribution of participants across occupational physical activity categories within strata of standard occupational code in women (n=139,529) and men (n=128,236) in UK Biobank. Figure S1. Flowchart detailing participant exclusions. Figure S2. Median, interquartile range, upper and lower adjacent values of average wrist acceleration in milli-g by occupational physical activity strata. Figure S3. Hazard ratio and 95% confidence intervals for association between non-occupational physical activity energy expenditure (PAEE) and all-cause mortality. Figure S4. Hazard ratio (HR) and 95% confidence interval (CI) of all-cause mortality by occupational group (Model 2). Figure S5. Hazard ratio (HR) and 95% confidence interval (CI) of all-cause mortality by occupational category excluding those with prevalent cardiovascular disease or cancer at baseline. Figure S6. Hazard ratio (HR) and 95% confidence interval (CI) of all-cause mortality by occupational category excluding paid workers with time in current job less than 10 years. Figure S7. Hazard ratio (HR) and 95% confidence interval (CI) of cardiovascular disease mortality (left) and cancer mortality (right) by occupational group (Model 2). Figure S8. Hazard ratio (HR) and 95% confidence interval (CI) of all-cause mortality by occupational group using Stan [file 12966_2021_1154_MOESM1_ESM.docx]

**Table S1** Creation of joint standing/walking work and manual work variable.

|  |  | Heavy manual or physical | | |
| --- | --- | --- | --- | --- |
|  |  | Never/rarely | Sometimes | Usually/always |
| Standing/walking | Never/rarely | Group 1 (n=91,406) | Group 1 (n=4,006) | Group 1  (n=582) |
|  | Sometimes | Group 2  (n=59,705) | Group 4  (n=20,459) | Group 6  (n=2,522) |
|  | Usually/always | Group 3  (n=25,469) | Group 5  (n=32,543) | Group 6  (n=31,073) |

All participants reporting never/rarely walking or standing at work were included in group 1 on the basis that it would be unlikely to be involved in any heavy manual or physical work without some standing/walking. There were few participants reporting sometimes walking or standing but usually/always being involved in heavy manual work, so these were included in group 6 on the basis that they would be similar to that group given their higher frequency of heavy manual work. Heavy manual or physical work from data field 816 (<http://biobank.ctsu.ox.ac.uk/crystal/field.cgi?id=816>), standing/walking work from data field 806 (<http://biobank.ctsu.ox.ac.uk/crystal/field.cgi?id=806>).

**Table S2** Standard occupational classifications (SOC) codes of paid workers in UK Biobank.

| First level SOC code | Types of occupation |
| --- | --- |
| Managers and senior officials | Corporate managers and senior officials |
|  | Production managers |
|  | Functional managers |
|  | Quality and customer care managers |
|  | Financial institution and office managers |
|  | Managers in distribution, storage and retailing |
|  | Protective service officers |
|  | Health and social services managers |
|  | Managers in farming, horticulture, forestry and fishing |
|  | Managers and proprietors in hospitality and leisure services |
|  | Managers and proprietors in other service industries |
| Professional occupations | Science professionals |
|  | Engineering professionals |
|  | Information and communication technology professionals |
|  | Health professionals |
|  | Teaching professionals |
|  | Research professionals |
|  | Legal professionals |
|  | Business and statistical professionals |
|  | Architects, town planners, surveyors |
|  | Public service professionals |
|  | Librarians and related professionals |
| Associate professional and technical occupations | Science and engineering technicians |
|  | Draughtspersons and building inspectors |
|  | IT service delivery occupations |
|  | Health associate professionals |
|  | Therapists |
|  | Social welfare associate professionals |
|  | Protective service occupations |
|  | Artistic and literary occupations |
|  | Design associate professionals |
|  | Media associate professionals |
|  | Sports and fitness occupations |
|  | Transport associate professionals |
|  | Legal associate professionals |
|  | Business and finance associate professionals |
|  | Sales and related associate professionals |
|  | Conservation associate professionals |
|  | Public service and other associate professionals |
| Administrative and secretarial occupations | Administrative occupations: government and related organisations |
|  | Administrative occupations: finance |
|  | Administrative occupations: records |
|  | Administrative occupations: communications |
|  | Administrative occupations: general |
|  | Secretarial and related occupations |
| Skilled trades occupations | Agricultural trades |
|  | Metal forming, welding and related trades |
|  | Metal machining, fitting and instrument making trades |
|  | Vehicle trades |
|  | Electrical trades |
|  | Construction trades |
|  | Building trades |
|  | Textiles and garments trades |
|  | Printing trades |
|  | Food preparation trades |
| Personal service occupations | Healthcare and related personal services |
|  | Childcare and related personal services |
|  | Animal care services |
|  | Leisure and travel service occupations |
|  | Hairdressers and related occupations |
|  | Housekeeping occupations |
| Sales and customer service occupations | Sales assistants and retail cashiers |
|  | Sales related occupations |
|  | Customer service occupations |
| Process, plant and machine operatives | Process operatives |
|  | Plant and machine operatives |
|  | Assemblers and routine operatives |
|  | Construction operatives |
|  | Transport drivers and operatives |
|  | Mobile machine drivers and operatives |
| Elementary occupations | Elementary agricultural occupations |
|  | Elementary construction occupations |
|  | Elementary process plant occupations |
|  | Elementary goods storage occupations |
|  | Elementary administration occupations |
|  | Elementary personal services occupations |
|  | Elementary cleaning occupations |
|  | Elementary security occupations |
|  | Elementary sales occupations |

Standard occupational classification codes from data field 20277 (<http://biobank.ctsu.ox.ac.uk/crystal/field.cgi?id=20277>)

**Table S3** Mutually adjusted sex-specific coefficients (standard errors) for prediction of average daily wrist acceleration (ln milli-g) from 13 self-reported behaviours.

| Variable | Women (n=52,507) | Men (n=40,918) |
| --- | --- | --- |
| Walking for pleasure (ln min/day) | .0227 (.0009) | .0177 (.0010) |
| Strenuous sports (ln min/day) | .0377 (.0014) | .0416 (.0014) |
| Other exercises (ln min/day) | .0242 (.0008) | .0280 (.0009) |
| Light DIY activities (min/day) | .0018 (.0009) | .0043 (.0010) |
| Heavy DIY activities (ln min/day) | .0156 (.0011) | .0163 (.0011) |
| Manual work (ln min/day) | .0120 (.0009) | .0189 (.0010) |
| Standing/walking at work (ln min/day) | .0099 (.0006) | .0095 (.0007) |
| Sedentary time at work (ln min/day) | .0039 (.0005) | .0048 (.0006) |
| Getting about method |  |  |
| Car or public transportation | Reference | Reference |
| Mixed use | .0080 (.0025) | .0112 (.0030) |
| Walking or cycling | .0360 (.0044) | .0502 (.0053) |
| Commuting method |  |  |
| Car or public transportation | Reference | Reference |
| Mixed use | .0366 (.0039) | .0391 (.0047) |
| Walking or cycling | .0522 (.0054) | .0539 (.0071) |
| TV viewing (hours/day) | -.0827 (.0026) | -.0817 (.0033) |
| Computer use (ln hours/day) | -.0609 (.0027) | -.0639 (.0030) |
| Sleep |  |  |
| ≤5.0 (hours/day) | -.0254 (.0064) | -.0080 (.0082) |
| 6.0 (hours/day) | -.0132 (.0032) | .0045 (.0038) |
| 7.0 (hours/day) | Reference | Reference |
| 8.0 (hours/day) | -.0206 (.0027) | -.0305 (.0034) |
| ≥9.0 (hours/day) | -.0735 (.0049) | -.0873 (.0064) |
| Constant | 3.378 (.0082) | 3.327 (.0102) |

To estimate non-occupational physical activity energy expenditure (PAEE), we used a method similar to one previously described [<https://doi.org/10.1186/s12966-020-00937-4>]. (1) Briefly, the previous method generated sex-specific regression models developed in the UK Biobank accelerometery sub-cohort to predict mean wrist acceleration from 14 self-reported behaviours. These self-reported behaviours and regression coefficients were then used to predict wrist acceleration in the main UK Biobank cohort. Predicted wrist acceleration was then converted to total daily PAEE in kJ/day/kg using data from a similarly aged UK cohort (2) [<https://dx.doi.org/10.1371%2Fjournal.pone.0167472>] and a previously reported scaling equation for dominant wrist acceleration (3) [<https://doi.org/10.1038/s41366-019-0352-x>].

For the present study, there were two adjustments to the above method for the slightly different purpose of predicting non-occupational PAEE. Firstly, we excluded moderate-to-vigorous physical activity (MVPA) from the prediction model because the questions used for this variable included both leisure and work activity, and so any subsequent prediction of non-occupational PAEE might include work related activity. This meant that 13 rather than 14 self-reported behaviours were used to generate the sex-specific regression models for predicting wrist acceleration, as shown above. The newly derived regression models and the original models including MVPA explained 14% and 17% variance in wrist acceleration for women and men respectively (i.e. there was no noticeable change in explanatory power when excluding MVPA).

Secondly, although still included in the prediction model, variables representing occupational physical activity (heavy manual work, standing/walking work, sedentary work) were set to zero at the prediction stage so that the resulting estimate represented non-occupational PAEE. Natural log transformations of self-reported behaviours used ln(x+1).

**Table S4** Baseline characteristics of women in paid employment in UK Biobank.

|  | No manual, no standing/walking | No manual, some standing/walking | No manual, usually standing/walking | Some manual, some standing/walking | Some manual, usually standing/walking | Usually manual, usually standing/walking |
| --- | --- | --- | --- | --- | --- | --- |
| n (%) | 52,385 | 31,485 | 16,464 | 9,034 | 17,093 | 13,068 |
| Age in years at baseline, mean (SD) | 52 (7) | 53 (7) | 53 (7) | 52 (7) | 53 (7) | 52 (7) |
| White ethnicity, % | 96 | 94 | 94 | 93 | 93 | 92 |
| Highest educational level |  |  |  |  |  |  |
| No qualification, % | 5 | 6 | 10 | 8 | 14 | 20 |
| Any other qualification, % | 53 | 50 | 44 | 57 | 60 | 63 |
| Degree or above, % | 42 | 44 | 46 | 34 | 25 | 17 |
| Townsend index, median (IQR) | -2.2 (-3.7;0.2) | -2.2 (-3.7;0.2) | -2.3 (-3.7;0.1) | -1.9 (-3.5;0.7) | -1.8 (-3.4;1.0) | -1.3 (-3.2;1.7) |
| Household income before tax |  |  |  |  |  |  |
| Prefer not to answer, % | 7 | 8 | 12 | 13 | 18 | 24 |
| Do not know, % | 19 | 20 | 20 | 24 | 25 | 27 |
| <£18,000, % | 29 | 30 | 29 | 29 | 26 | 22 |
| £18,000-£30,999, % | 29 | 27 | 24 | 20 | 15 | 10 |
| £31,000-£51,999, % | 8 | 6 | 4 | 3 | 2 | 2 |
| £52,000-£100,000, % | 7 | 8 | 8 | 9 | 9 | 10 |
| >£100,000, % | 1 | 2 | 3 | 3 | 4 | 5 |
| Smoking status |  |  |  |  |  |  |
| Never, % | 60 | 61 | 65 | 60 | 60 | 57 |
| Previous, % | 31 | 30 | 27 | 30 | 28 | 29 |
| Current, % | 8 | 8 | 8 | 10 | 12 | 14 |
| Alcohol use status |  |  |  |  |  |  |
| Never, % | 3 | 4 | 5 | 4 | 6 | 7 |
| Previous, % | 2 | 2 | 3 | 3 | 3 | 4 |
| Current, % | 95 | 94 | 92 | 93 | 91 | 89 |
| Fruit/vegetable score, median (IQR) | 2 (1;2) | 2 (1;3) | 2 (1;3) | 2 (1;3) | 2 (1;3) | 2 (1;3) |
| Red/processed meat score, median (IQR) | 1 (1;1) | 1 (1;1) | 1 (1;1) | 1 (1;1) | 1 (1;1) | 1 (1;1) |
| Adds salt to food, % | 27 | 28 | 26 | 30 | 29 | 29 |
| Consumes oily fish, % | 37 | 35 | 35 | 33 | 35 | 33 |
| Non-work PAEE (kJ/kg/day), mean (SD) | 44 (3) | 44 (3) | 44 (3) | 44 (3) | 44 (3) | 44 (3) |
| Working hours per week, median (IQR) | 35 (24;40) | 35 (24;40) | 30 (18;40) | 35 (24;40) | 30 (20;38) | 30 (20;38) |
| Job involves shift work |  |  |  |  |  |  |
| Never/rarely, % | 95 | 91 | 87 | 78 | 69 | 59 |
| Sometimes, % | 3 | 6 | 5 | 13 | 12 | 11 |
| Usually/always, % | 2 | 3 | 8 | 9 | 19 | 29 |
| Years in current paid job, median (IQR) | 9 (4;18) | 10 (4;18) | 10 (5;20) | 10 (5;19) | 10 (4;19) | 9 (4;18) |
| Parental history of CVD or cancer, % | 68 | 70 | 69 | 69 | 69 | 68 |
| Blood pressure or cholesterol medication, % | 13 | 15 | 15 | 14 | 16 | 16 |
| Diagnosis of diabetes or insulin prescription, % | 2 | 2 | 2 | 3 | 3 | 3 |
| Baseline CVD, % | 6 | 7 | 7 | 7 | 8 | 8 |
| Baseline cancer, % | 7 | 7 | 8 | 8 | 7 | 7 |
| Resting heart rate in bpm, mean (SD) | 70 (10) | 70 (10) | 70 (10) | 70 (10) | 70 (10) | 70 (10) |
| Body mass index |  |  |  |  |  |  |
| <25 kg/m2, % | 45 | 43 | 45 | 40 | 40 | 40 |
| 25-30 kg/m2, % | 34 | 36 | 36 | 35 | 37 | 36 |
| >30 kg/m2, % | 21 | 22 | 19 | 25 | 24 | 25 |

bpm=beats per minute; CVD=cardiovascular disease; IQR=interquartile range; PAEE=physical activity energy expenditure; SD=standard deviation.

**Table S5** Baseline characteristics of men in paid employment in UK Biobank.

|  | No manual, no standing/walking | No manual, some standing/walking | No manual, usually standing/walking | Some manual, some standing/walking | Some manual, usually standing/walking | Usually manual, usually standing/walking |
| --- | --- | --- | --- | --- | --- | --- |
| n (%) | 43,609 | 28,220 | 9,005 | 11,425 | 15,450 | 20,527 |
| Age in years at baseline, mean (SD) | 53 (7) | 54 (7) | 54 (7) | 53 (7) | 54 (7) | 53 (7) |
| White ethnicity, % | 95 | 95 | 91 | 94 | 92 | 94 |
| Highest educational level |  |  |  |  |  |  |
| No qualification, % | 4 | 4 | 10 | 14 | 18 | 26 |
| Any other qualification, % | 42 | 45 | 44 | 61 | 62 | 63 |
| Degree or above, % | 53 | 50 | 46 | 25 | 19 | 10 |
| Townsend index, median (IQR) | -2.5 (-3.9;-0.1) | -2.5 (-3.9;-0.3) | -2.1 (-3.6;0.5) | -2.1 (-3.6;0.5) | -1.8 (-3.4;1.0) | -1.5 (-3.2;1.4) |
| Household income before tax |  |  |  |  |  |  |
| Prefer not to answer, % | 3 | 3 | 8 | 8 | 12 | 15 |
| Do not know, % | 12 | 12 | 20 | 22 | 29 | 34 |
| <£18,000, % | 27 | 30 | 32 | 35 | 32 | 29 |
| £18,000-£30,999, % | 39 | 38 | 28 | 24 | 16 | 10 |
| £31,000-£51,999, % | 14 | 11 | 6 | 3 | 2 | 1 |
| £52,000-£100,000, % | 4 | 5 | 5 | 7 | 7 | 9 |
| >£100,000, % | 1 | 1 | 1 | 1 | 2 | 3 |
| Smoking status |  |  |  |  |  |  |
| Never, % | 58 | 56 | 55 | 51 | 49 | 48 |
| Previous, % | 33 | 34 | 33 | 36 | 36 | 34 |
| Current, % | 10 | 9 | 11 | 13 | 15 | 17 |
| Alcohol use status |  |  |  |  |  |  |
| Never, % | 2 | 2 | 3 | 2 | 3 | 3 |
| Previous, % | 2 | 2 | 3 | 3 | 3 | 3 |
| Current, % | 96 | 96 | 93 | 95 | 94 | 94 |
| Fruit/vegetable score, median (IQR) | 1 (1;2) | 1 (1;2) | 1 (1;2) | 1 (0;2) | 1 (0;2) | 1 (0;2) |
| Red/processed meat score, median (IQR) | 1 (1;1) | 1 (1;1) | 1 (1;1) | 1 (1;1) | 1 (1;1) | 1 (1;1) |
| Adds salt to food, % | 27 | 29 | 27 | 31 | 31 | 31 |
| Consumes oily fish, % | 39 | 37 | 38 | 37 | 36 | 36 |
| Non-work PAEE (kJ/kg/day), mean (SD) | 43 (4) | 43 (4) | 43 (4) | 43 (4) | 43 (4) | 43 (4) |
| Working hours per week, median (IQR) | 40 (36;45) | 40 (37;45) | 40 (30;45) | 40 (37;48) | 40 (36;45) | 40 (37;48) |
| Job involves shift work |  |  |  |  |  |  |
| Never/rarely, % | 91 | 88 | 80 | 71 | 65 | 66 |
| Sometimes, % | 4 | 6 | 7 | 15 | 13 | 14 |
| Usually/always, % | 5 | 5 | 13 | 14 | 22 | 20 |
| Years in current paid job, median (IQR) | 10 (4;20) | 11 (5;22) | 10 (4;22) | 12 (5;23) | 10 (4;22) | 12 (5;25) |
| Parental history of CVD or cancer, % | 66 | 68 | 67 | 66 | 66 | 65 |
| Blood pressure or cholesterol medication, % | 22 | 25 | 26 | 24 | 25 | 22 |
| Diagnosis of diabetes or insulin prescription, % | 4 | 5 | 6 | 5 | 5 | 4 |
| Baseline CVD, % | 8 | 10 | 12 | 10 | 12 | 10 |
| Baseline cancer, % | 5 | 5 | 5 | 5 | 5 | 4 |
| Resting heart rate in bpm, mean (SD) | 68 (11) | 68 (11) | 68 (12) | 68 (11) | 68 (11) | 68 (11) |
| Body mass index |  |  |  |  |  |  |
| <25 kg/m2, % | 28 | 25 | 25 | 21 | 24 | 24 |
| 25-30 kg/m2, % | 49 | 51 | 51 | 50 | 50 | 50 |
| >30 kg/m2, % | 23 | 24 | 24 | 30 | 27 | 26 |

bpm=beats per minute; CVD=cardiovascular disease; IQR=interquartile range; PAEE=physical activity energy expenditure; SD=standard deviation

**Table S6** Baseline characteristics of women not in paid employment in UK Biobank.

|  | Retired | Caring for home/family | Unable to work due to illness | Unemployed | Unpaid work | Student |
| --- | --- | --- | --- | --- | --- | --- |
| n (%) | 89,224 | 11,872 | 6,078 | 2,573 | 1,560 | 807 |
| Age in years at baseline, mean (SD) | 64 (4) | 52 (7) | 53 (6) | 51 (6) | 57 (7) | 48 (6) |
| White ethnicity, % | 97 | 91 | 91 | 80 | 92 | 78 |
| Highest educational level |  |  |  |  |  |  |
| No qualification, % | 27 | 16 | 32 | 19 | 10 | 5 |
| Any other qualification, % | 48 | 53 | 50 | 52 | 46 | 43 |
| Degree or above, % | 23 | 30 | 16 | 27 | 44 | 51 |
| Townsend index, median (IQR) | -2.5 (-3.8;-0.3) | -2.4 (-3.8;0.3) | 0.8 (-2.3;3.7) | 0.4 (-2.4;3.5) | -2.0 (-3.7;0.7) | -0.4 (-2.8;3.0) |
| Household income before tax |  |  |  |  |  |  |
| Prefer not to answer, % | 32 | 17 | 50 | 47 | 20 | 37 |
| Do not know, % | 26 | 15 | 17 | 13 | 16 | 14 |
| <£18,000, % | 13 | 17 | 9 | 11 | 15 | 15 |
| £18,000-£30,999, % | 5 | 17 | 3 | 6 | 13 | 13 |
| £31,000-£51,999, % | 1 | 11 | 1 | 2 | 9 | 3 |
| £52,000-£100,000, % | 15 | 13 | 9 | 11 | 17 | 10 |
| >£100,000, % | 9 | 9 | 12 | 10 | 9 | 7 |
| Smoking status |  |  |  |  |  |  |
| Never, % | 58 | 64 | 46 | 55 | 62 | 61 |
| Previous, % | 35 | 27 | 29 | 25 | 29 | 29 |
| Current, % | 6 | 8 | 24 | 19 | 8 | 10 |
| Alcohol use status |  |  |  |  |  |  |
| Never, % | 6 | 8 | 11 | 12 | 7 | 8 |
| Previous, % | 4 | 4 | 13 | 5 | 4 | 5 |
| Current, % | 90 | 88 | 75 | 83 | 89 | 87 |
| Fruit/vegetable score, median (IQR) | 2 (1;3) | 2 (1;3) | 1 (1;2) | 1 (1;2) | 2 (1;3) | 2 (1;3) |
| Red/processed meat score, median (IQR) | 1 (1;1) | 1 (1;1) | 1 (1;1) | 1 (1;1) | 1 (1;1) | 1 (1;1) |
| Adds salt to food, % | 28 | 28 | 27 | 27 | 26 | 29 |
| Consumes oily fish, % | 27 | 33 | 31 | 33 | 29 | 34 |
| Non-work PAEE (kJ/kg/day), mean (SD) | 43 (3) | 44 (3) | 41 (3) | 43 (3) | 44 (3) | 43 (4) |
| Working hours per week, median (IQR) | 0 | 0 | 0 | 0 | 0 | 0 |
| Job involves shift work |  |  |  |  |  |  |
| Never/rarely, % | 0 | 0 | 0 | 0 | 0 | 0 |
| Sometimes, % | 0 | 0 | 0 | 0 | 0 | 0 |
| Usually/always, % | 0 | 0 | 0 | 0 | 0 | 0 |
| Years in current paid job, median (IQR) | 0 | 0 | 0 | 0 | 0 | 0 |
| Parental history of CVD or cancer, % | 77 | 68 | 75 | 65 | 74 | 61 |
| Blood pressure or cholesterol medication, % | 36 | 16 | 36 | 18 | 20 | 10 |
| Diagnosis of diabetes or insulin prescription, % | 5 | 4 | 10 | 5 | 3 | 2 |
| Baseline CVD, % | 13 | 7 | 21 | 8 | 9 | 7 |
| Baseline cancer, % | 13 | 7 | 13 | 8 | 9 | 5 |
| Resting heart rate in bpm, mean (SD) | 71 (11) | 71 (11) | 74 (12) | 71 (11) | 70 (11) | 70 (10) |
| Body mass index |  |  |  |  |  |  |
| <25 kg/m2, % | 35 | 47 | 26 | 33 | 49 | 44 |
| 25-30 kg/m2, % | 41 | 32 | 30 | 35 | 32 | 35 |
| >30 kg/m2, % | 24 | 21 | 44 | 31 | 19 | 21 |

bpm=beats per minute; CVD=cardiovascular disease; IQR=interquartile range; PAEE=physical activity energy expenditure; SD=standard deviation

**Table S7** Baseline characteristics of men not in paid employment in UK Biobank.

|  | Retired | Caring for home/family | Unable to work due to illness | Unemployed | Unpaid work | Student |
| --- | --- | --- | --- | --- | --- | --- |
| n (%) | 66,653 | 1,100 | 7,494 | 4,777 | 597 | 401 |
| Age in years at baseline, mean (SD) | 64 (4) | 52 (7) | 56 (7) | 53 (7) | 56 (8) | 48 (6) |
| White ethnicity, % | 97 | 86 | 93 | 86 | 87 | 68 |
| Highest educational level |  |  |  |  |  |  |
| No qualification, % | 24 | 19 | 44 | 23 | 9 | 6 |
| Any other qualification, % | 44 | 49 | 43 | 51 | 40 | 32 |
| Degree or above, % | 30 | 31 | 12 | 25 | 50 | 61 |
| Townsend index, median (IQR) | -2.6 (-3.86;-.4) | -0.0 (-2.8;3.0) | 1.6 (-1.6;4.4) | 0.6 (-2.5;3.9) | -0.3 (-2.9;2.8) | 1.1 (-2.1;4.3) |
| Household income before tax |  |  |  |  |  |  |
| Prefer not to answer, % | 30 | 40 | 64 | 53 | 37 | 41 |
| Do not know, % | 30 | 18 | 11 | 14 | 17 | 18 |
| <£18,000, % | 19 | 13 | 4 | 9 | 14 | 14 |
| £18,000-£30,999, % | 7 | 10 | 2 | 5 | 10 | 7 |
| £31,000-£51,999, % | 1 | 4 | 0 | 1 | 5 | 2 |
| £52,000-£100,000, % | 10 | 8 | 8 | 9 | 11 | 8 |
| >£100,000, % | 2 | 8 | 10 | 9 | 6 | 10 |
| Smoking status |  |  |  |  |  |  |
| Never, % | 43 | 43 | 30 | 43 | 50 | 59 |
| Previous, % | 48 | 34 | 38 | 31 | 31 | 25 |
| Current, % | 9 | 23 | 31 | 25 | 18 | 15 |
| Alcohol use status |  |  |  |  |  |  |
| Never, % | 2 | 6 | 4 | 5 | 4 | 15 |
| Previous, % | 3 | 7 | 13 | 6 | 7 | 9 |
| Current, % | 94 | 87 | 82 | 89 | 88 | 76 |
| Fruit/vegetable score, median (IQR) | 1 (1;2) | 1 (0;2) | 1 (0;2) | 1 (0;2) | 1 (1;2) | 1 (1;2) |
| Red/processed meat score, median (IQR) | 1 (1;1) | 1 (1;1) | 1 (1;2) | 1 (1;1) | 1 (1;1) | 1 (1;2) |
| Adds salt to food, % | 27 | 29 | 29 | 30 | 28 | 28 |
| Consumes oily fish, % | 30 | 35 | 31 | 33 | 32 | 35 |
| Non-work PAEE (kJ/kg/day), mean (SD) | 42 (3) | 42 (4) | 40 (3) | 41 (3) | 42 (3) | 42 (4) |
| Working hours per week, median (IQR) | 0 | 0 | 0 | 0 | 0 | 0 |
| Job involves shift work |  |  |  |  |  |  |
| Never/rarely, % | 0 | 0 | 0 | 0 | 0 | 0 |
| Sometimes, % | 0 | 0 | 0 | 0 | 0 | 0 |
| Usually/always, % | 0 | 0 | 0 | 0 | 0 | 0 |
| Years in current paid job, median (IQR) | 0 | 0 | 0 | 0 | 0 | 0 |
| Parental history of CVD or cancer, % | 73 | 68 | 71 | 64 | 65 | 55 |
| Blood pressure or cholesterol medication, % | 49 | 25 | 53 | 29 | 29 | 15 |
| Diagnosis of diabetes or insulin prescription, % | 9 | 7 | 16 | 9 | 6 | 5 |
| Baseline CVD, % | 22 | 15 | 35 | 13 | 12 | 7 |
| Baseline cancer, % | 11 | 4 | 10 | 5 | 7 | 2 |
| Resting heart rate in bpm, mean (SD) | 68 (12) | 70 (12) | 74 (14) | 71 (13) | 69 (12) | 70 (11) |
| Body mass index |  |  |  |  |  |  |
| <25 kg/m2, % | 25 | 30 | 21 | 27 | 31 | 30 |
| 25-30 kg/m2, % | 51 | 44 | 38 | 44 | 47 | 50 |
| >30 kg/m2, % | 24 | 26 | 41 | 28 | 22 | 20 |

bpm=beats per minute; CVD=cardiovascular disease; IQR=interquartile range; PAEE=physical activity energy expenditure; SD=standard deviation

**Table S8** Distribution of participants across occupational physical activity categories within strata of standard occupational code in women (n=139,529) and men (n=128,236) in UK Biobank.

|  |  | Occupational physical activity category | | | | | |  |
| --- | --- | --- | --- | --- | --- | --- | --- | --- |
| Sex | Standard occupational code strata | No manual, no standing/walking | No manual, some standing/walking | No manual, usually standing/walking | Some manual, some standing/walking | Some manual, usually standing/walking | Usually manual, usually standing/walking | Total |
| Women | Managers and Senior Officials | 9,455 (54%) | 4,083 (23%) | 893 (5%) | 1,051 (6%) | 1281 (7%) | 833 (5%) | 17,596 (100%) |
|  | Professional | 10,263 (34%) | 8,877 (29%) | 6,697 (22%) | 1,393 (5%) | 2,598 (9%) | 586 (2%) | 30,414 (100%) |
|  | Associate Professional and Technical | 10,128 (36%) | 6,399 (23%) | 2,492 (9%) | 2,392 (8%) | 3,938 (14%) | 2,933 (10%) | 28,282 (100%) |
|  | Administrative and Secretarial | 19,385 (58%) | 8,570 (26%) | 1,730 (5%) | 2,014 (6%) | 1213 (4%) | 482 (1%) | 33,394 (100%) |
|  | Skilled Trades | 151 (7%) | 139 (6%) | 159 (7%) | 175 (8%) | 673 (30%) | 973 (43%) | 2,270 (100%) |
|  | Personal Care and Service | 906 (7%) | 2,263 (17%) | 2,337 (17%) | 1,311 (10%) | 3,554 (26%) | 3,099 (23%) | 13,470 (100%) |
|  | Sales and Customer Service | 1,434 (21%) | 765 (11%) | 1,170 (17%) | 323 (5%) | 2,027 (29%) | 1,229 (18%) | 6,948 (100%) |
|  | Process, Plant and Machine Operatives | 413 (30%) | 174 (12%) | 112 (8%) | 180 (13%) | 216 (16%) | 298 (21%) | 1,393 (100%) |
|  | Elementary | 250 (4%) | 215 (4%) | 874 (15%) | 195 (3%) | 1,593 (28%) | 2,635 (46%) | 5,762 (100%) |
|  |  |  |  |  |  |  |  |  |
| Men | Managers and Senior Officials | 12,394 (44%) | 7,692 (27%) | 1,396 (5%) | 2,774 (10%) | 2,560 (9%) | 1,480 (5%) | 28,296 (100%) |
|  | Professional | 14,107 (43%) | 10,749 (33%) | 3,607 (11%) | 1,685 (5%) | 1,843 (6%) | 677 (2%) | 32,668 (100%) |
|  | Associate Professional and Technical | 7,511 (39%) | 5,083 (26%) | 1,463 (8%) | 2,070 (11%) | 2,118 (11%) | 1,177 (6%) | 19,422 (100%) |
|  | Administrative and Secretarial | 4,487 (53%) | 1,896 (23%) | 449 (5%) | 812 (10%) | 474 (6%) | 291 (3%) | 8,409 (100%) |
|  | Skilled Trades | 1,062 (6%) | 809 (5%) | 418 (2%) | 1,489 (9%) | 3,920 (23%) | 9,423 (55%) | 17,121 (100%) |
|  | Personal Care and Service | 203 (7%) | 362 (12%) | 431 (15%) | 325 (11%) | 841 (29%) | 749 (26%) | 2,911 (100%) |
|  | Sales and Customer Service | 539 (23%) | 326 (14%) | 282 (12%) | 174 (8%) | 535 (23%) | 464 (20%) | 2,320 (100%) |
|  | Process, Plant and Machine Operatives | 2,988 (29%) | 821 (8%) | 328 (3%) | 1,585 (15%) | 1,456 (14%) | 3,125 (30%) | 10,303 (100%) |
|  | Elementary | 318 (5%) | 482 (7%) | 631 (9%) | 511 (8%) | 1,703 (25%) | 3,141 (46%) | 6,786 (100%) |

| 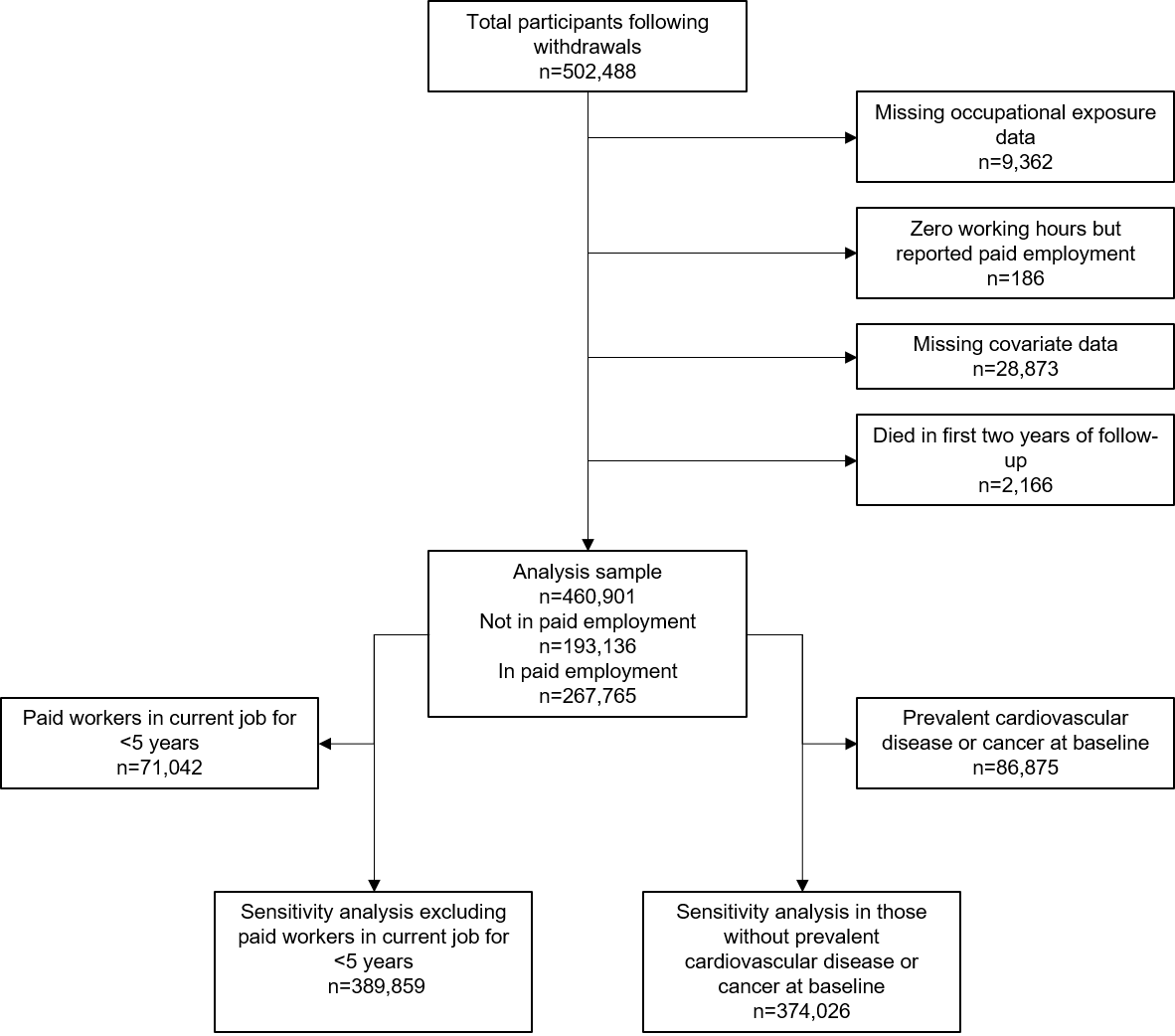 |
| --- |
| **Figure S1** Flowchart detailing participant exclusions. |

| 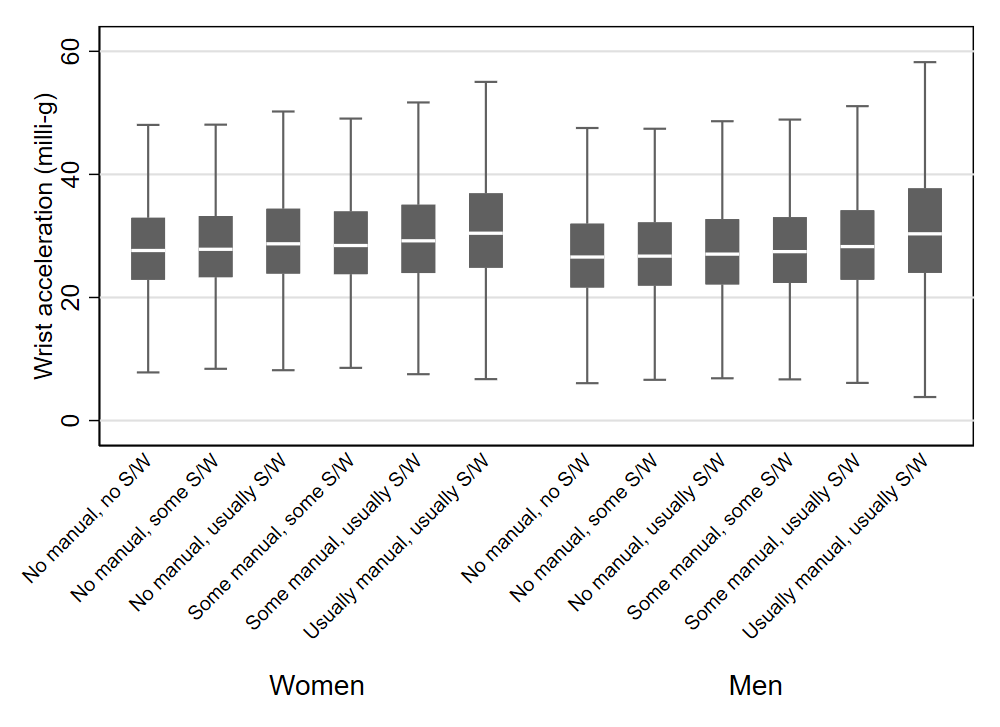 |
| --- |
| **Figure S2** Median, interquartile range, upper and lower adjacent values of average wrist acceleration in milli-g by occupational physical activity strata. Data are for those participants in the UK Biobank accelerometery sub-cohort with valid accelerometer data and in paid employment at baseline (n=63,512).  S/W=standing/walking.  Physical activity assessment using accelerometers occurred a median 5.7 years after baseline assessment of occupational physical activity variables. The collection and processing of the accelerometer data have been described in greater detail previously (4). Between 2013 and 2015 invitations to participate in the accelerometer sub-cohort were sent to 236,519 participants who had provided a valid email address at recruitment. Consenting participants (n=106,053) were sent an accelerometer (Axivity AX3, Newcastle upon Tyne, UK) initialised to capture three-dimensional acceleration at 100 Hertz continuously for seven days which they were asked to begin wearing immediately on their dominant wrist. Euclidean norm minus one (ENMO) was calculated as the Euclidean norm (vector magnitude) of calibrated acceleration (5) in three axes minus one gravitational unit (1,000 milli-g) and negative values were truncated to zero (6). Periods of ≥60 minutes during which the standard deviations of all three axes were <13.0 milli-g were identified as non-wear. Mean wrist ENMO in milli-g was summarised across valid wear-time (data across the full 24-hour spectrum and at least 72 hours of wear in total) for each individual whilst minimising diurnal bias caused by non-wear (7). |

| 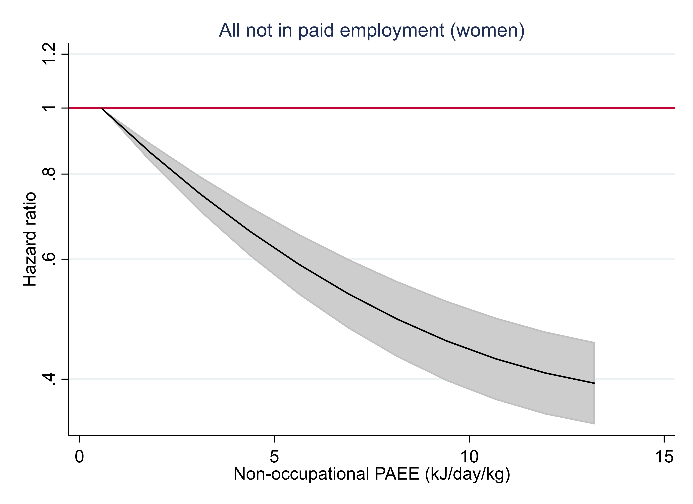 | 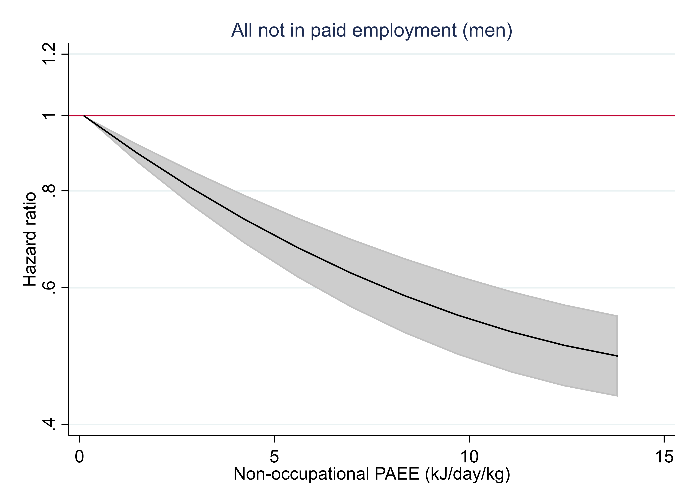 |
| --- | --- |
| 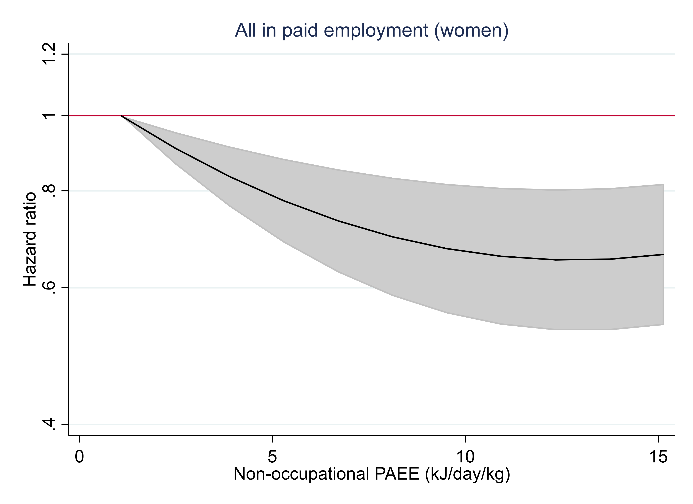 | 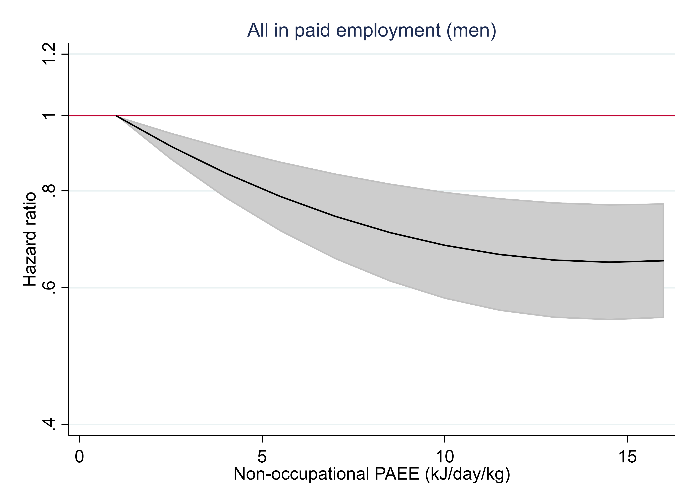 |
| 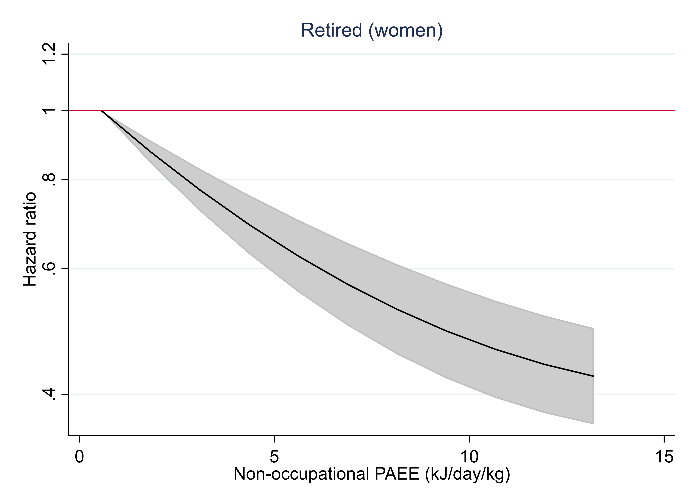 | 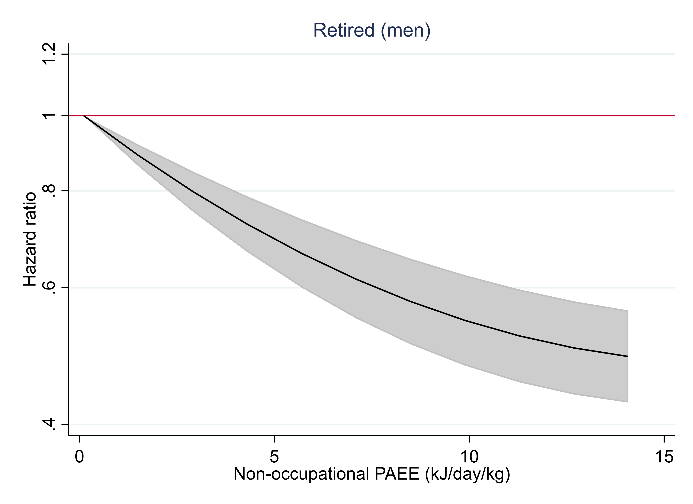 |
| 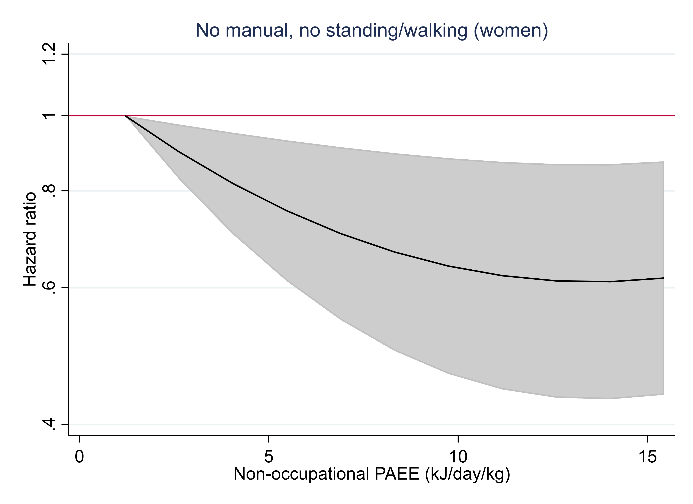 | 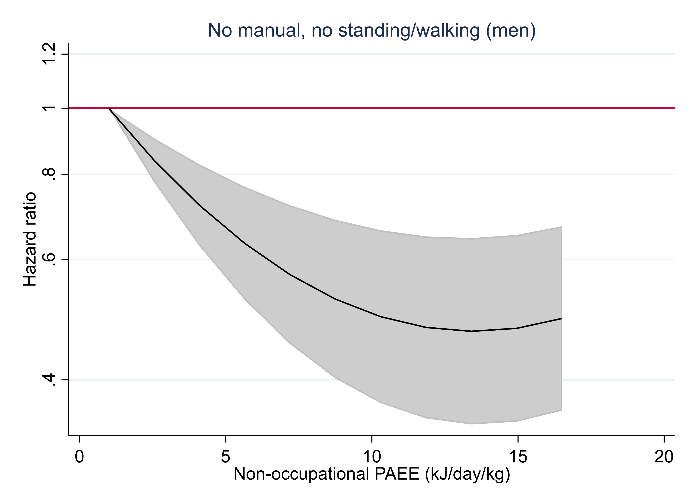 |
| 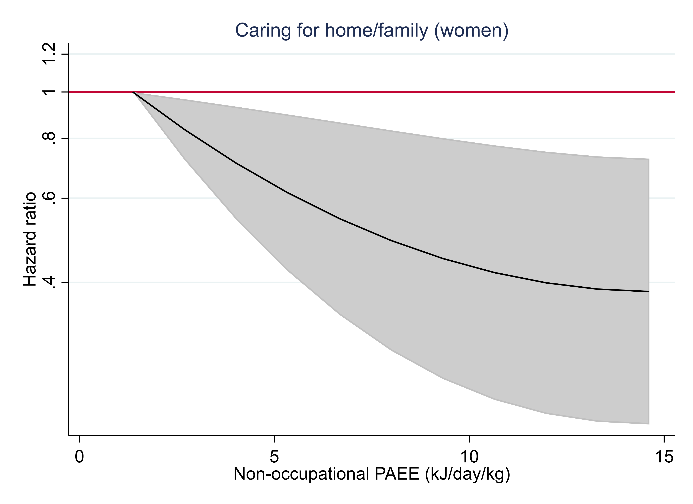 | 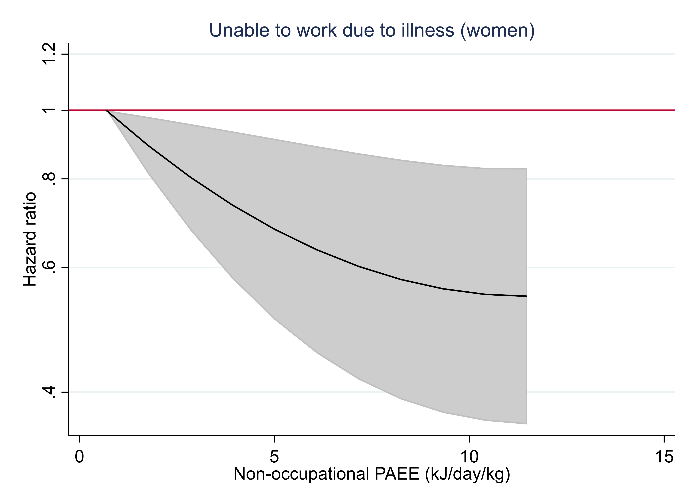 |
| 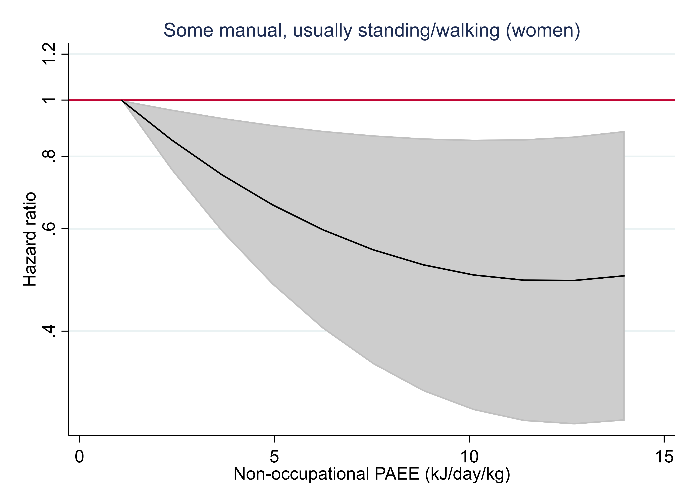 | 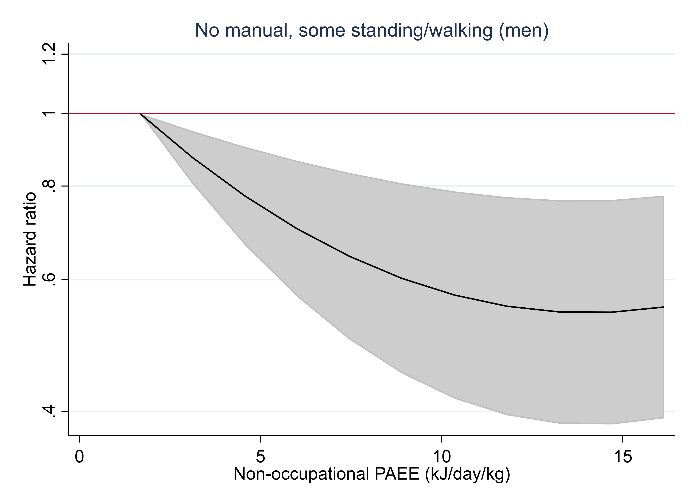 |
| **Figure S3** Hazard ratio and 95% confidence intervals for association between non-occupational physical activity energy expenditure (PAEE) and all-cause mortality. Separate results are presented for all non-working and working women and men, as well as in subgroups for which the quadratic non-occucpational PAEE term was statistically significant (p<0.05). The exposure is rescaled to the level of non-occupational PAEE above the least active participant in the overall sample. The x-axis range is limited to 5-95^th^ percentiles of the exposure in those who died with a reference level of the least active participant in each subgroup (denoted by horizontal y-axis line at 1). Model 2 hazard ratios are adjusted for age (underlying timescale), ethnicity, Townsend deprivation index, highest educational level (stratified baseline hazard), annual household income (stratified baseline hazard), working hours per week, years in current job, job involves shift work, alcohol consumption, smoking, salt added to food, oily fish intake, fruit and vegetable intake (stratified baseline hazard), processed and red meat intake, non-occupational physical activity energy expenditure, parental history of cancer or cardiovascular disease, use of blood pressure or cholesterol lowering medications, doctor-diagnosed diabetes or treatment with insulin, baseline prevalent cancer, baseline prevalent cardiovascular disease, body mass index, resting heart rate. | |

| 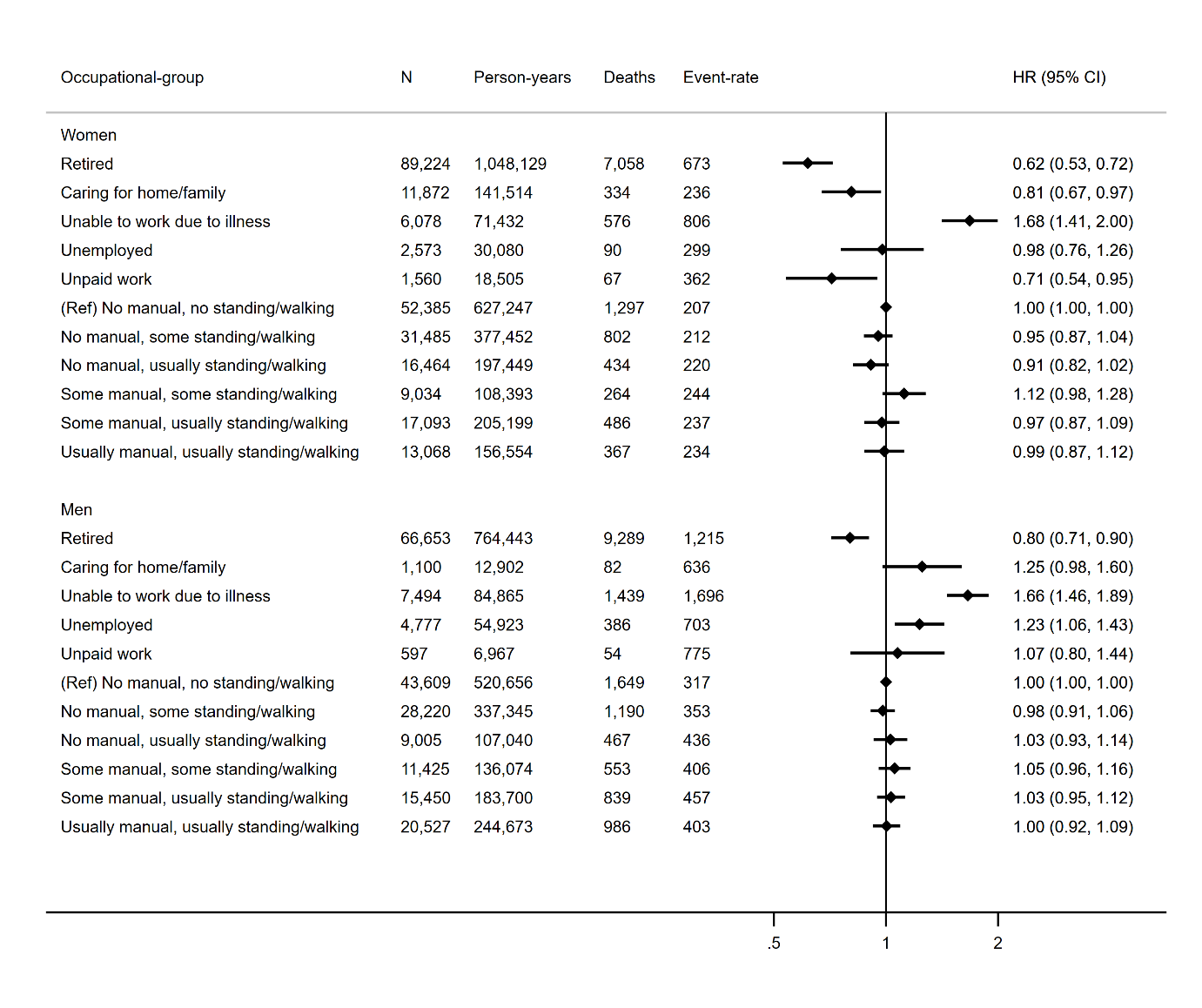 |
| --- |
| **Figure S4** Hazard ratio (HR) and 95% confidence interval (CI) of all-cause mortality by occupational group. Reference group is “no manual, no standing/walking”. Model 2 hazard ratios are adjusted for age (underlying timescale), ethnicity, Townsend deprivation index, highest educational level (stratified baseline hazard), annual household income (stratified baseline hazard), working hours per week, years in current job, job involves shift work, alcohol consumption, smoking, salt added to food, oily fish intake, fruit and vegetable intake (stratified baseline hazard), processed and red meat intake, non-occupational physical activity energy expenditure, parental history of cancer or cardiovascular disease, use of blood pressure or cholesterol lowering medications, doctor-diagnosed diabetes or treatment with insulin, baseline prevalent cancer, baseline prevalent cardiovascular disease, body mass index, resting heart rate. Results for students not shown due to small numbers of events. Event-rate per 100,000 person-years. |

| 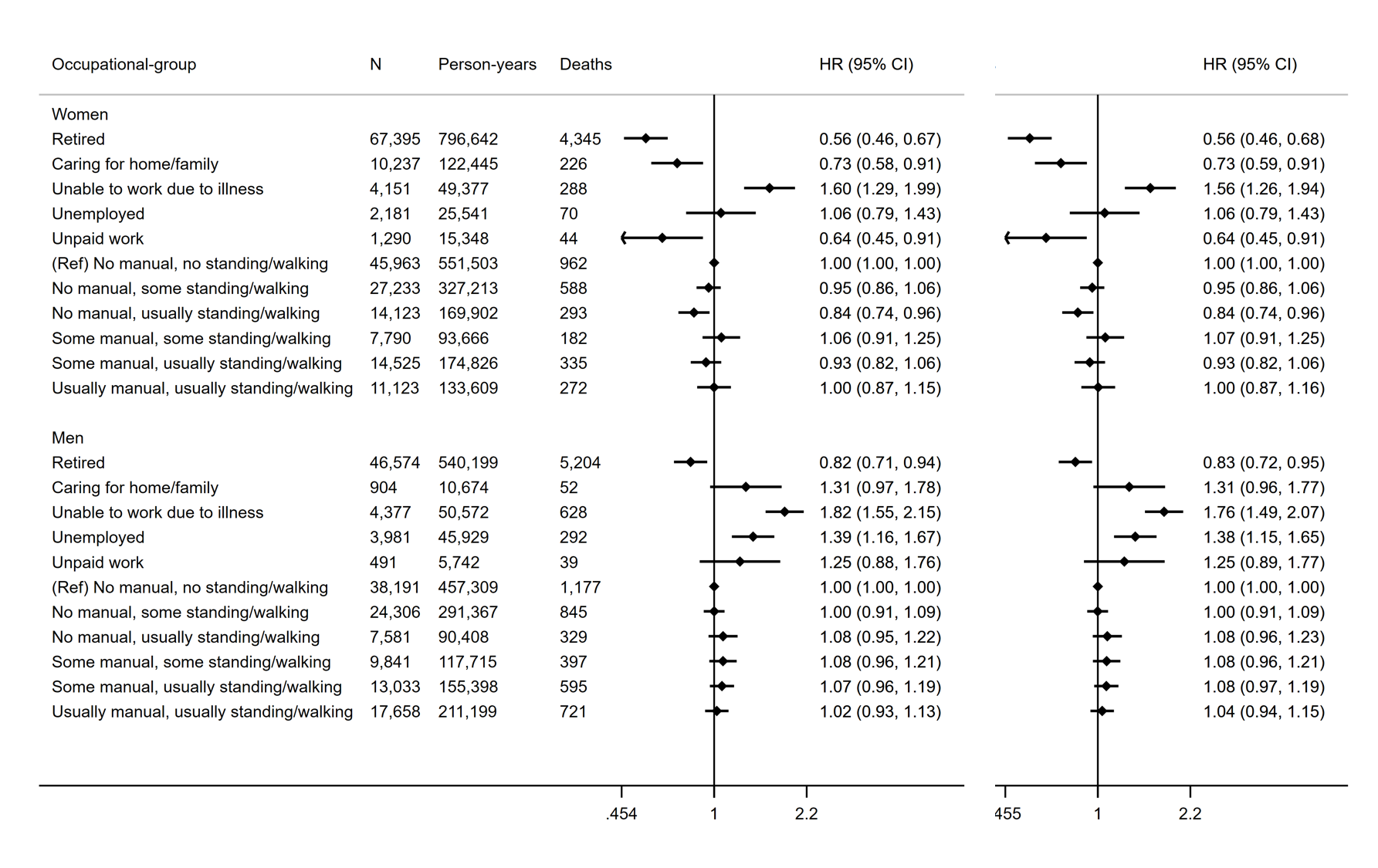 |
| --- |
| **Figure S5** Hazard ratio (HR) and 95% confidence interval (CI) of all-cause mortality by occupational category excluding those with prevalent cardiovascular disease or cancer at baseline. Reference group is “no manual, no standing/walking”. Model 1 (left) hazard ratios are adjusted for age (underlying timescale), ethnicity, Townsend deprivation index, highest educational level (stratified baseline hazard), annual household income (stratified baseline hazard), working hours per week, years in current job, job involves shift work, alcohol consumption, smoking, salt added to food, oily fish intake, fruit and vegetable intake (stratified baseline hazard), processed and red meat intake, non-occupational physical activity energy expenditure, parental history of cancer or cardiovascular disease, use of blood pressure or cholesterol lowering medications, doctor-diagnosed diabetes or treatment with insulin. Model 2 (right) hazard ratios are additionally adjusted for body mass index, resting heart rate. Arrow indicates confidence interval boundary out of range. Results for students not shown due to small numbers of events. |
| 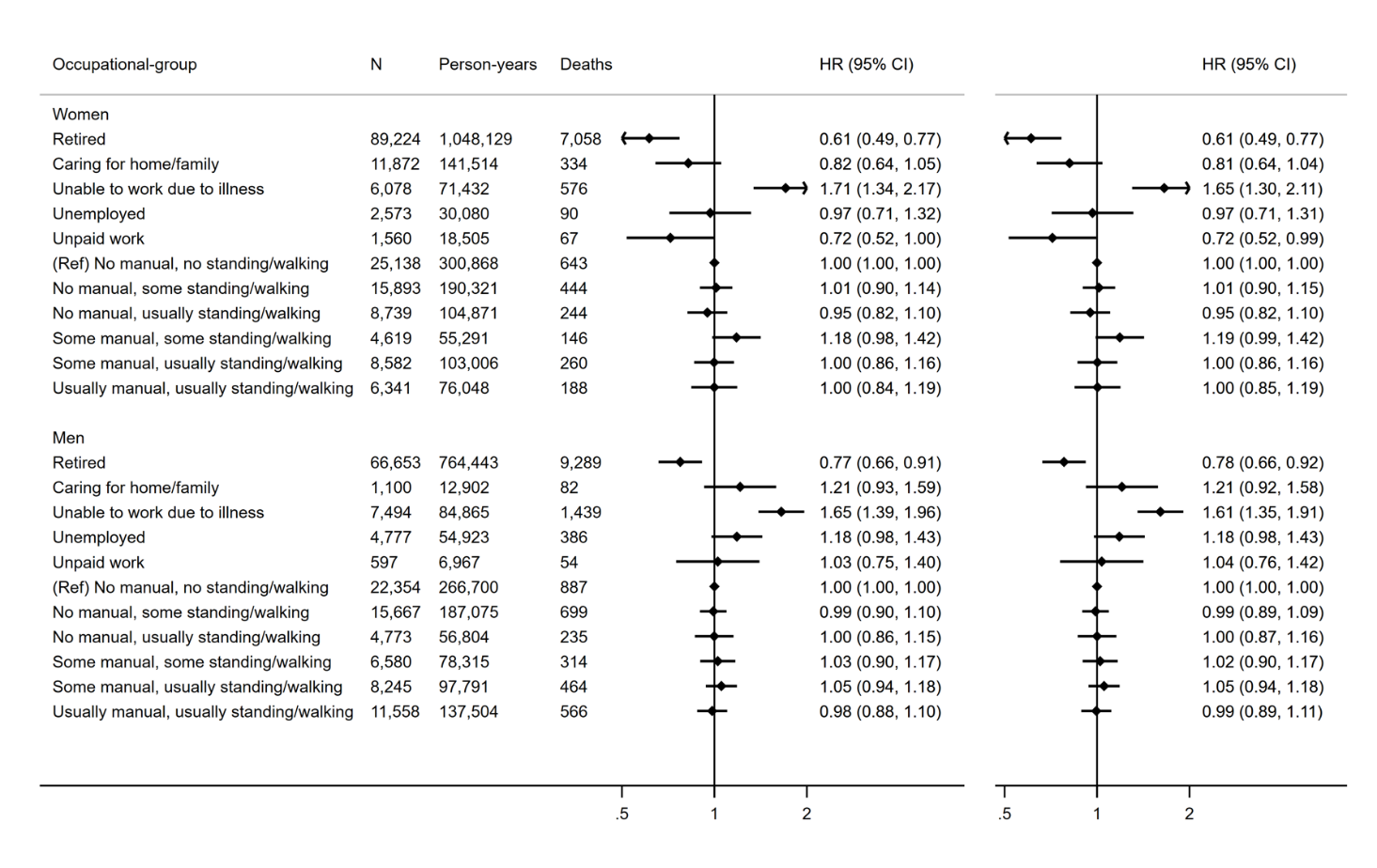 |
| **Figure S6** Hazard ratio (HR) and 95% confidence interval (CI) of all-cause mortality by occupational category excluding paid workers with time in current job less than 10 years. Reference group is “no manual, no standing/walking”. Model 1 (left) hazard ratios are adjusted for age (underlying timescale), ethnicity, Townsend deprivation index, highest educational level (stratified baseline hazard), annual household income (stratified baseline hazard), working hours per week, years in current job, job involves shift work, alcohol consumption, smoking, salt added to food, oily fish intake, fruit and vegetable intake (stratified baseline hazard), processed and red meat intake, non-occupational physical activity energy expenditure, parental history of cancer or cardiovascular disease, use of blood pressure or cholesterol lowering medications, doctor-diagnosed diabetes or treatment with insulin, baseline prevalent cancer, baseline prevalent cardiovascular disease. Model 2 (right) hazard ratios are additionally adjusted for body mass index, resting heart rate. Arrow indicates confidence interval boundary out of range. Results for students not shown due to small numbers of events. |
| 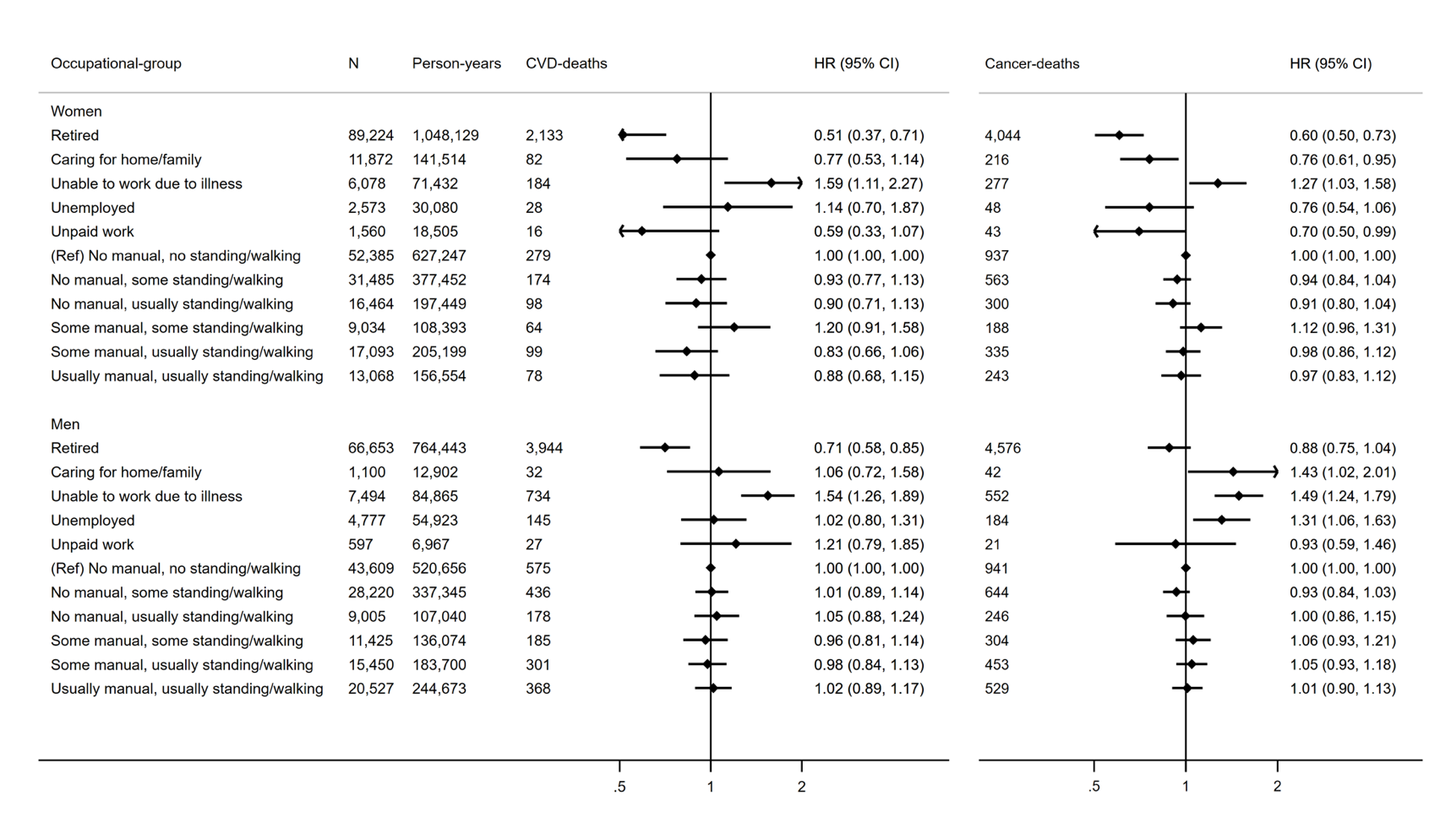 |
| **Figure S7** Hazard ratio (HR) and 95% confidence interval (CI) of cardiovascular disease mortality (left) and cancer mortality (right) by occupational group. Reference group is “no manual, no standing/walking”. Model 2 hazard ratios are adjusted for age (underlying timescale), ethnicity, Townsend deprivation index, highest educational level (stratified baseline hazard), annual household income (stratified baseline hazard), working hours per week, years in current job, job involves shift work, alcohol consumption, smoking, salt added to food, oily fish intake, fruit and vegetable intake (stratified baseline hazard), processed and red meat intake, non-occupational physical activity energy expenditure, parental history of cancer or cardiovascular disease, use of blood pressure or cholesterol lowering medications, doctor-diagnosed diabetes or treatment with insulin, baseline prevalent cancer, baseline prevalent cardiovascular disease, body mass index, resting heart rate. Arrow indicates confidence interval boundary out of range. |
| 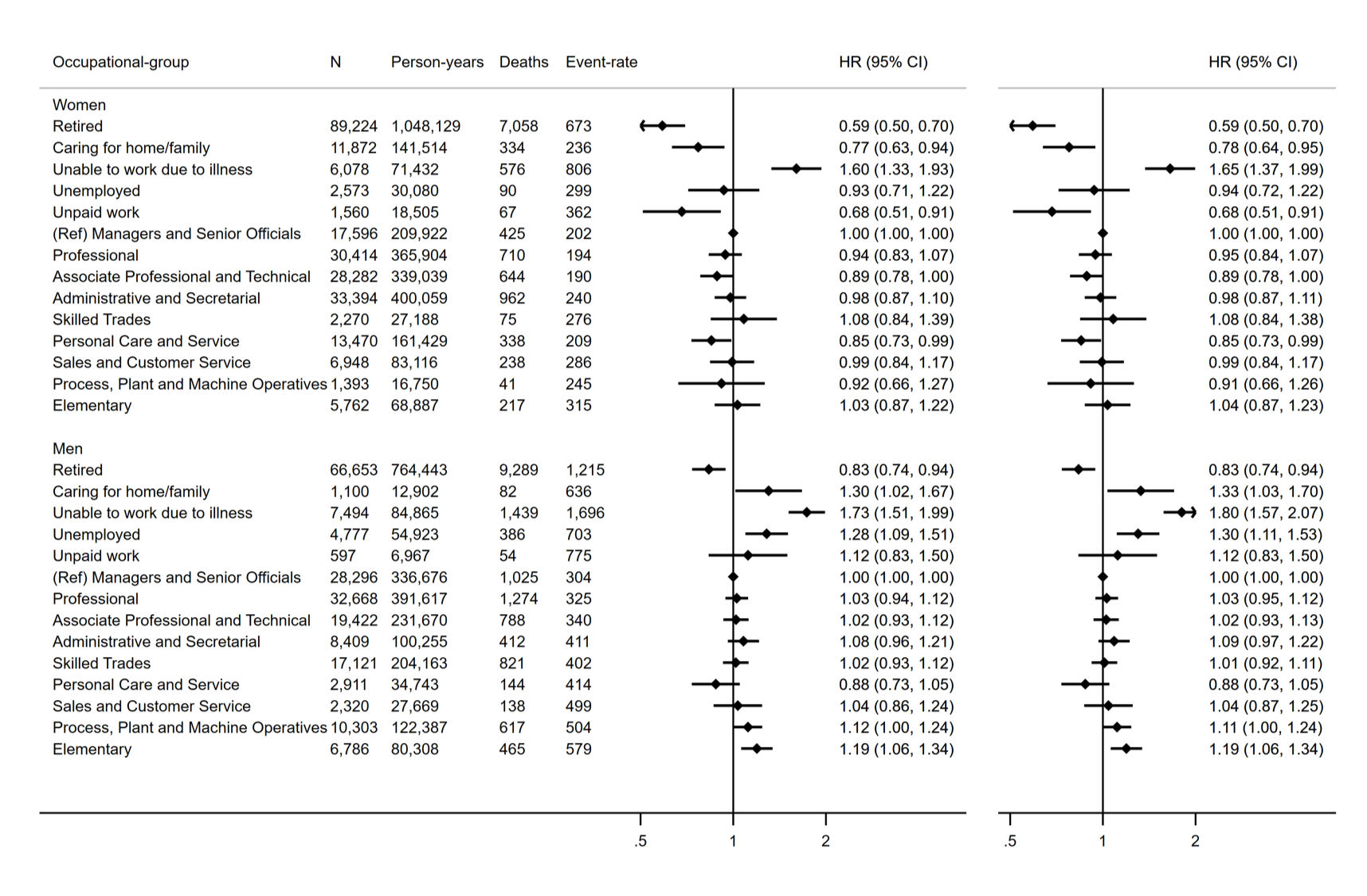 |
| **Figure S8** Hazard ratio (HR) and 95% confidence interval (CI) of all-cause mortality by occupational group. Reference group is “Managers and Senior Officials”. Model 1 (left) hazard ratios are adjusted for age (underlying timescale), ethnicity, Townsend deprivation index, highest educational level (stratified baseline hazard), annual household income (stratified baseline hazard), working hours per week, years in current job, job involves shift work, alcohol consumption, smoking, salt added to food, oily fish intake, fruit and vegetable intake (stratified baseline hazard), processed and red meat intake, non-occupational physical activity energy expenditure, parental history of cancer or cardiovascular disease, use of blood pressure or cholesterol lowering medications, doctor-diagnosed diabetes or treatment with insulin, baseline prevalent cancer, baseline prevalent cardiovascular disease. Model 2 (right) hazard ratios are additionally adjusted for body mass index, resting heart rate. Arrow indicates confidence interval boundary out of range. Results for students not shown due to small numbers of events. Event-rate per 100,000 person-years. |
| 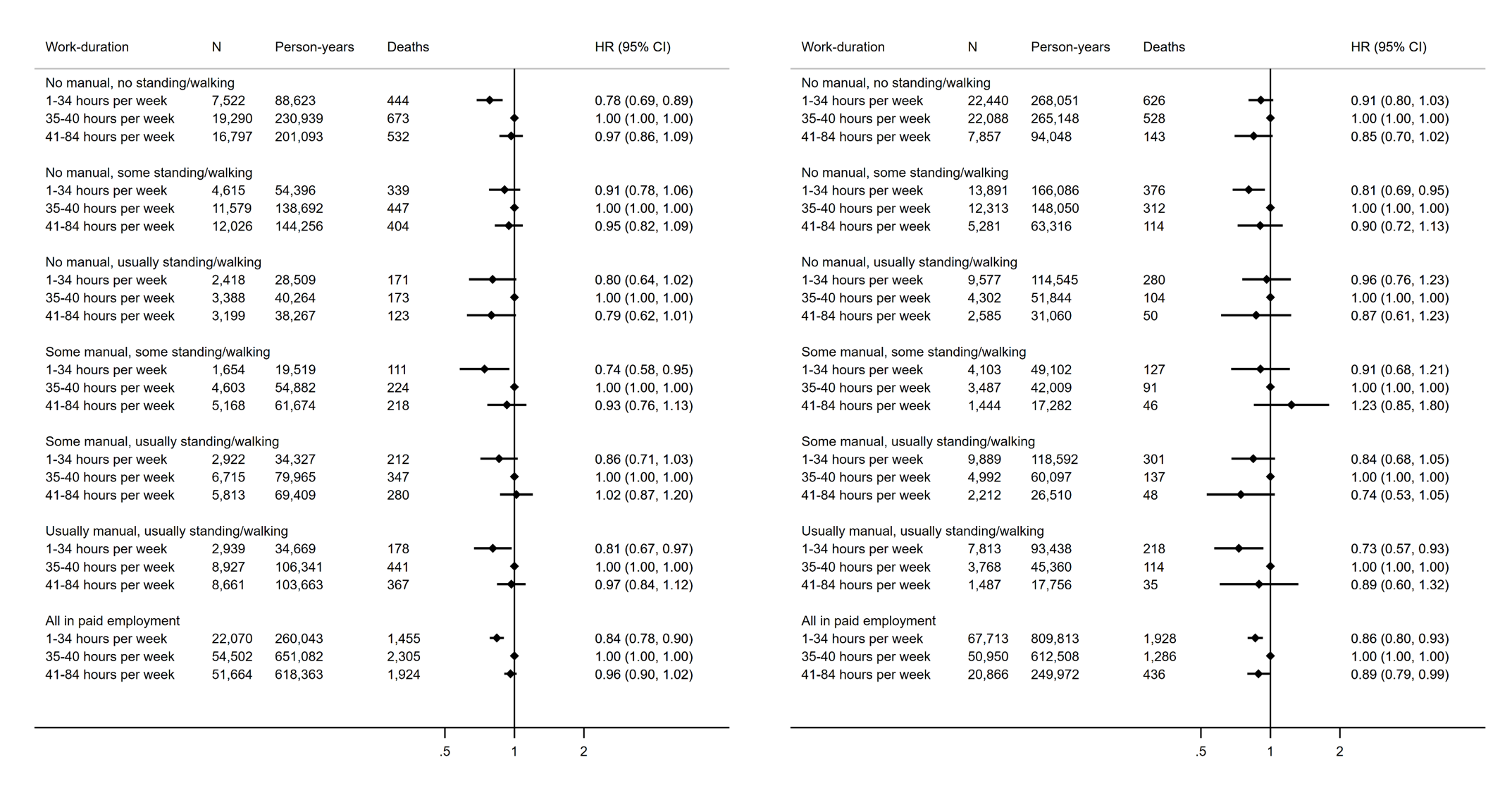 |
| **Figure S9** Hazard ratio (HR) and 95% confidence interval (CI) of all-cause mortality by tertile of work duration in hours per week across occupational physical activity strata in women (left) and men (right) in paid employment. Reference group is “35-40 hours per week”. Model 2 hazard ratios are adjusted for age (underlying timescale), ethnicity, Townsend deprivation index, highest educational level (stratified baseline hazard), annual household income (stratified baseline hazard), years in current job, job involves shift work, alcohol consumption, smoking, salt added to food, oily fish intake, fruit and vegetable intake (stratified baseline hazard), processed and red meat intake, non-occupational physical activity energy expenditure, parental history of cancer or cardiovascular disease, use of blood pressure or cholesterol lowering medications, doctor-diagnosed diabetes or treatment with insulin, baseline prevalent cancer, baseline prevalent cardiovascular disease, body mass index, resting heart rate. Arrow indicates confidence interval boundary out of range. |

| 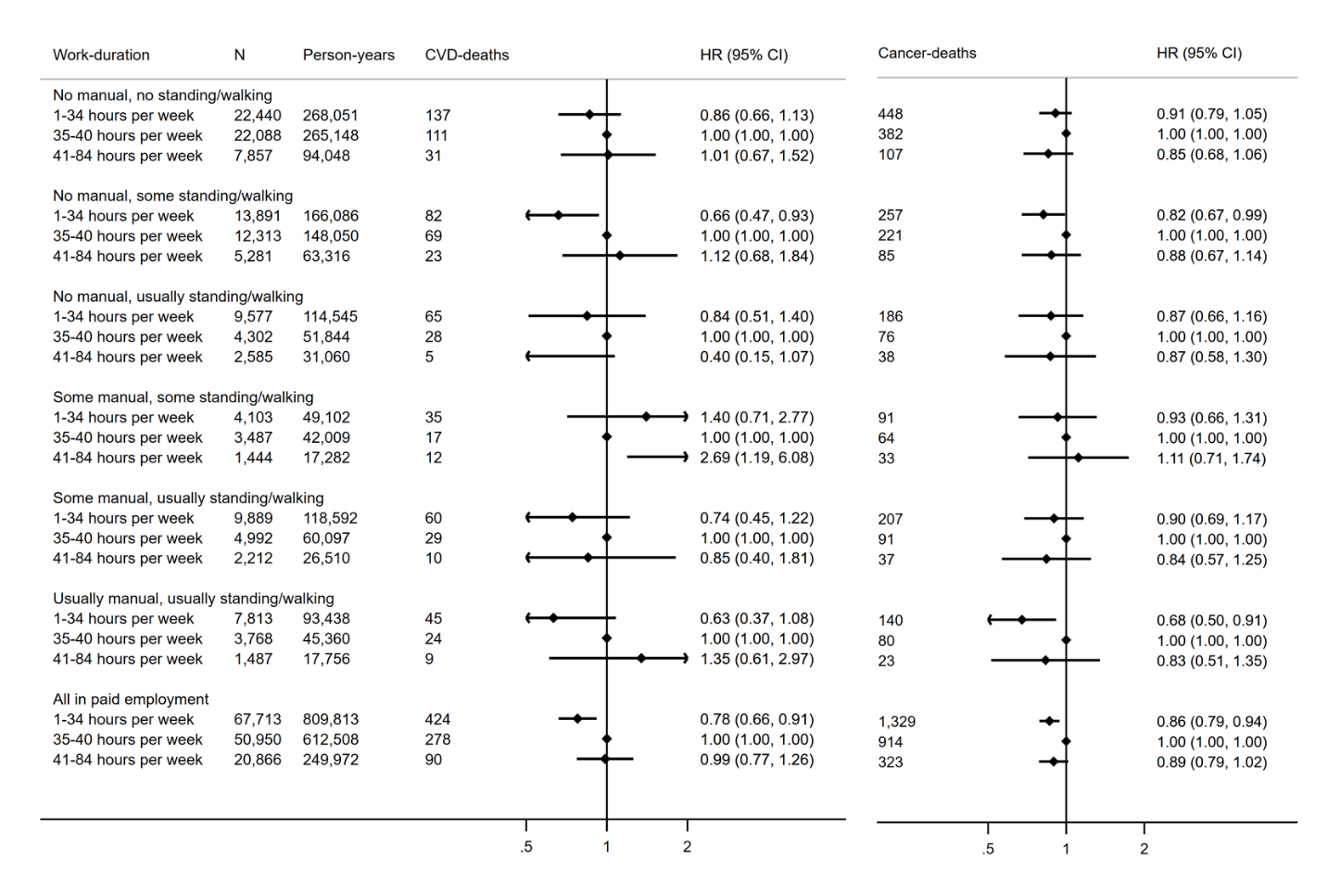 |
| --- |
| **Figure S10** Hazard ratio (HR) and 95% confidence interval (CI) of cardiovascular disease mortality (left) and cancer mortality (right) by tertile of work duration in hours per week across occupational physical activity strata for women in paid employment. Reference group is “35-40 hours per week”. Model 2 hazard ratios are adjusted for age (underlying timescale), ethnicity, Townsend deprivation index, highest educational level (stratified baseline hazard), annual household income (stratified baseline hazard), years in current job, job involves shift work, alcohol consumption, smoking, salt added to food, oily fish intake, fruit and vegetable intake (stratified baseline hazard), processed and red meat intake, non-occupational physical activity energy expenditure, parental history of cancer or cardiovascular disease, use of blood pressure or cholesterol lowering medications, doctor-diagnosed diabetes or treatment with insulin, baseline prevalent cancer, baseline prevalent cardiovascular disease, body mass index, resting heart rate. Arrow indicates confidence interval boundary out of range. Results for “No manual, usually standing/walking” not shown due to small numbers of events. Interaction of work duration by occupational physical activity strata: cardiovascular disease (p=0.51), cancer (0.05). |
| 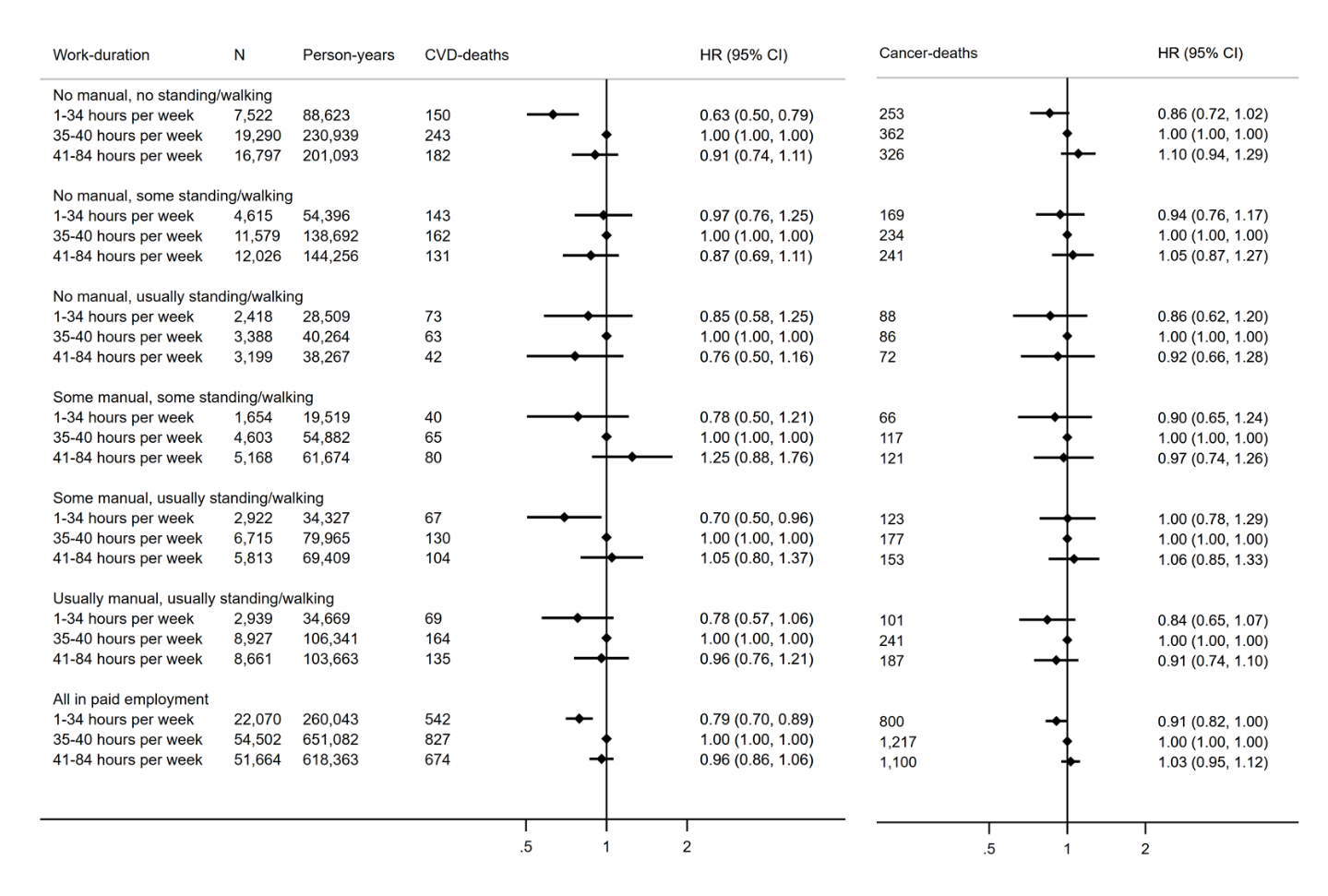 |
| **Figure S11** Hazard ratio (HR) and 95% confidence interval (CI) of cardiovascular disease mortality (left) and cancer mortality (right) by tertile of work duration in hours per week across occupational physical activity strata for men in paid employment. Reference group is “35-40 hours per week”. Model 2 hazard ratios are adjusted for age (underlying timescale), ethnicity, Townsend deprivation index, highest educational level (stratified baseline hazard), annual household income (stratified baseline hazard), years in current job, job involves shift work, alcohol consumption, smoking, salt added to food, oily fish intake, fruit and vegetable intake (stratified baseline hazard), processed and red meat intake, non-occupational physical activity energy expenditure, parental history of cancer or cardiovascular disease, use of blood pressure or cholesterol lowering medications, doctor-diagnosed diabetes or treatment with insulin, baseline prevalent cancer, baseline prevalent cardiovascular disease, body mass index, resting heart rate. Arrow indicates confidence interval boundary out of range. Results for “No manual, usually standing/walking” not shown due to small numbers of events. Interaction of work duration by occupational physical activity strata: cardiovascular disease (p=0.77), cancer (0.90). |

| **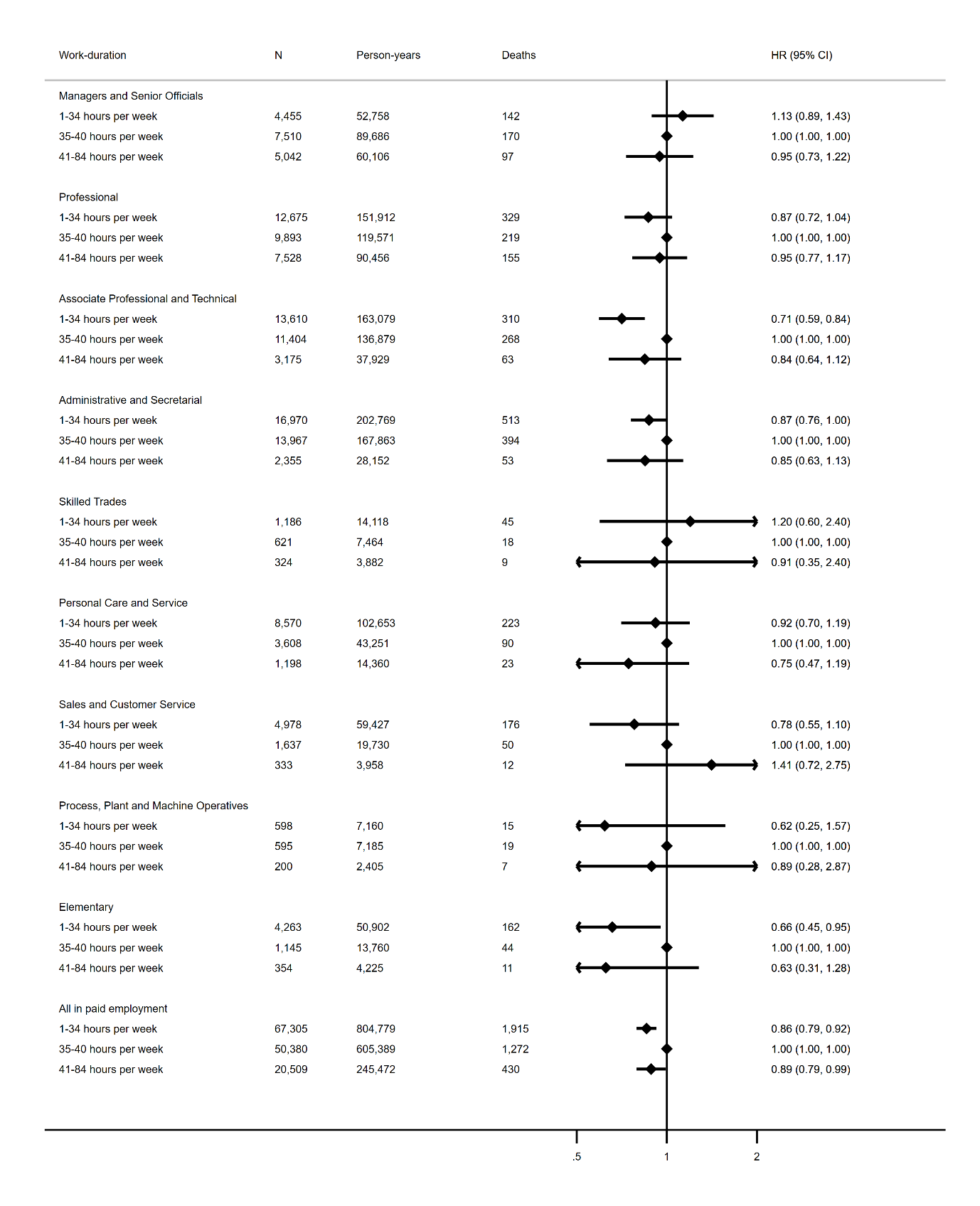** |
| --- |
| **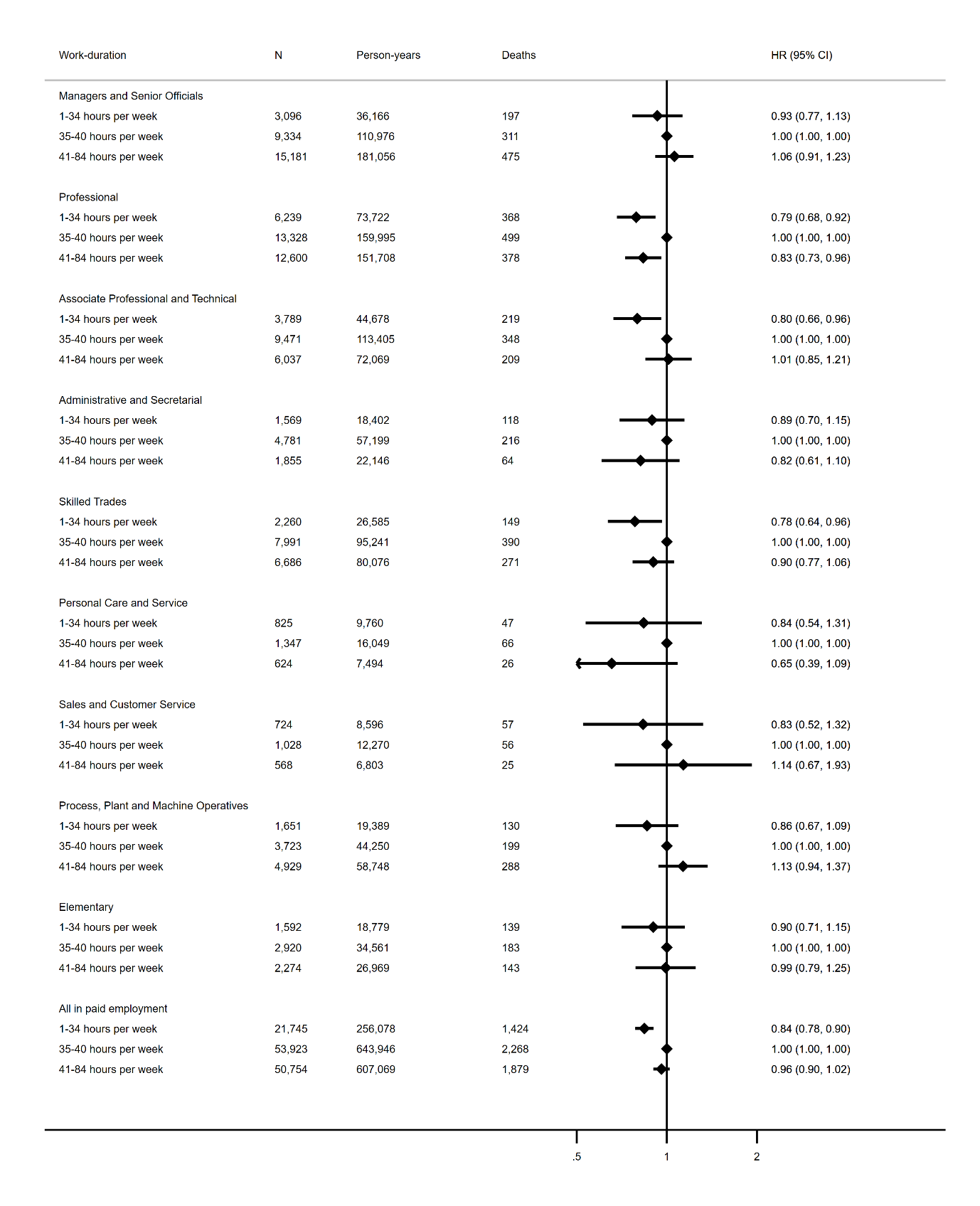** |
| **Figure S12** Hazard ratio (HR) and 95% confidence interval (CI) of all-cause mortality by tertile of work duration in hours per week across standard occupational code strata for women (top) and men (bottom) in paid employment. Reference group is “35-40 hours per week”. Model 2 hazard ratios are adjusted for age (underlying timescale), ethnicity, Townsend deprivation index, highest educational level (stratified baseline hazard), annual household income (stratified baseline hazard), years in current job, job involves shift work, alcohol consumption, smoking, salt added to food, oily fish intake, fruit and vegetable intake (stratified baseline hazard), processed and red meat intake, non-occupational physical activity energy expenditure, parental history of cancer or cardiovascular disease, use of blood pressure or cholesterol lowering medications, doctor-diagnosed diabetes or treatment with insulin, baseline prevalent cancer, baseline prevalent cardiovascular disease, body mass index, resting heart rate. Arrow indicates confidence interval boundary out of range. |

| 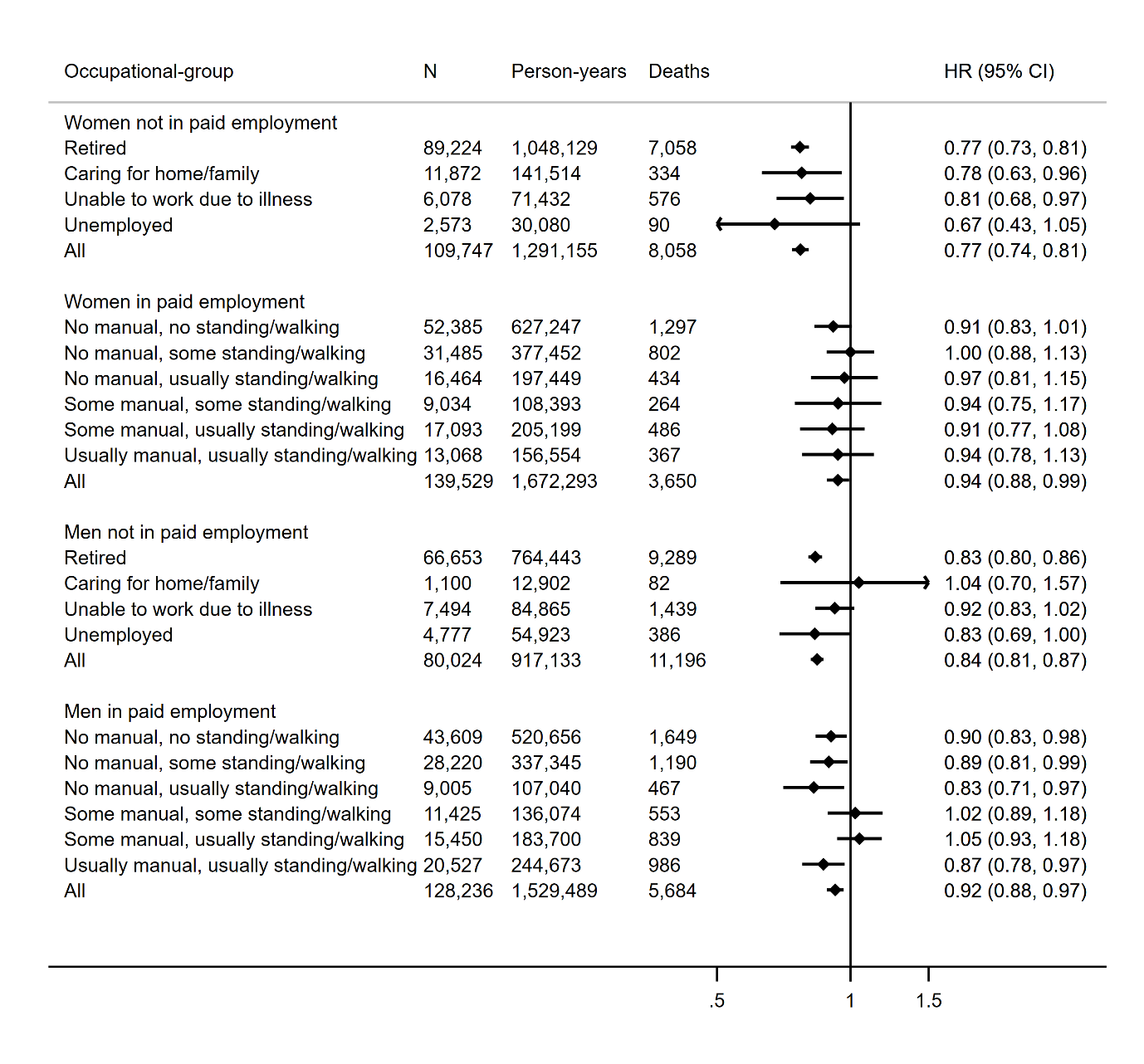 |
| --- |
| **Figure S13** Hazard ratio (HR) and 95% confidence interval (CI) of all-cause mortality per 5 kJ/day/kg of non-occupational physical activity energy expenditure across occupational strata. Model 2 hazard ratios are adjusted for age (underlying timescale), ethnicity, Townsend deprivation index, highest educational level (stratified baseline hazard), annual household income (stratified baseline hazard), working hours per week, years in current job, job involves shift work, alcohol consumption, smoking, salt added to food, oily fish intake, fruit and vegetable intake (stratified baseline hazard), processed and red meat intake, parental history of cancer or cardiovascular disease, use of blood pressure or cholesterol lowering medications, doctor-diagnosed diabetes or treatment with insulin, baseline prevalent cancer, baseline prevalent cardiovascular disease, body mass index, resting heart rate. Arrow indicates confidence interval boundary out of range. Results for students and unpaid workers not shown due to small numbers of events. |

| 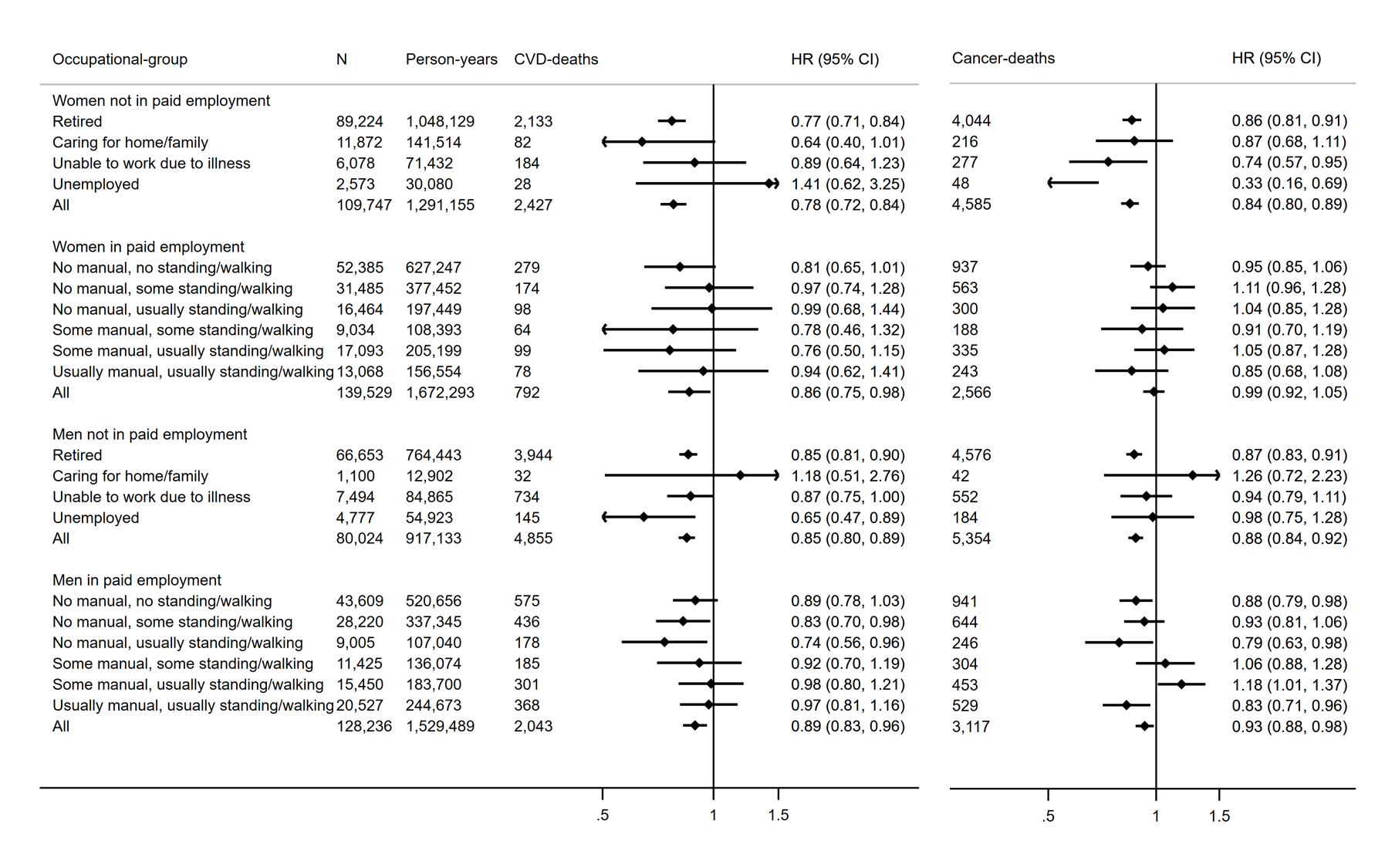 |
| --- |
| **Figure S14** Hazard ratio (HR) and 95% confidence interval (CI) of cardiovascular disease mortality (left) and cancer mortality (right) per 5 kJ/day/kg of non-occupational physical activity energy expenditure across occupational strata. Model 2 hazard ratios are adjusted for age (underlying timescale), ethnicity, Townsend deprivation index, highest educational level (stratified baseline hazard), annual household income (stratified baseline hazard), working hours per week, years in current job, job involves shift work, alcohol consumption, smoking, salt added to food, oily fish intake, fruit and vegetable intake (stratified baseline hazard), processed and red meat intake, parental history of cancer or cardiovascular disease, use of blood pressure or cholesterol lowering medications, doctor-diagnosed diabetes or treatment with insulin, baseline prevalent cancer, baseline prevalent cardiovascular disease, body mass index, resting heart rate. Results for students and unpaid workers not shown due to small numbers of events. Arrow indicates confidence interval boundary out of range. Interaction of non-occupational physical activity energy expenditure by occupational strata for those in paid employment: cardiovascular disease in women in (p=0.37), cancer in women (0.32), cardiovascular disease in men (p=0.70), cancer in men (0.05). Interaction of non-occupational physical activity energy expenditure by occupational strata for those not in paid employment: cardiovascular disease in women in (p=0.70), cancer in women (0.04), cardiovascular disease in men (p=0.77), cancer in men (0.23). |

| 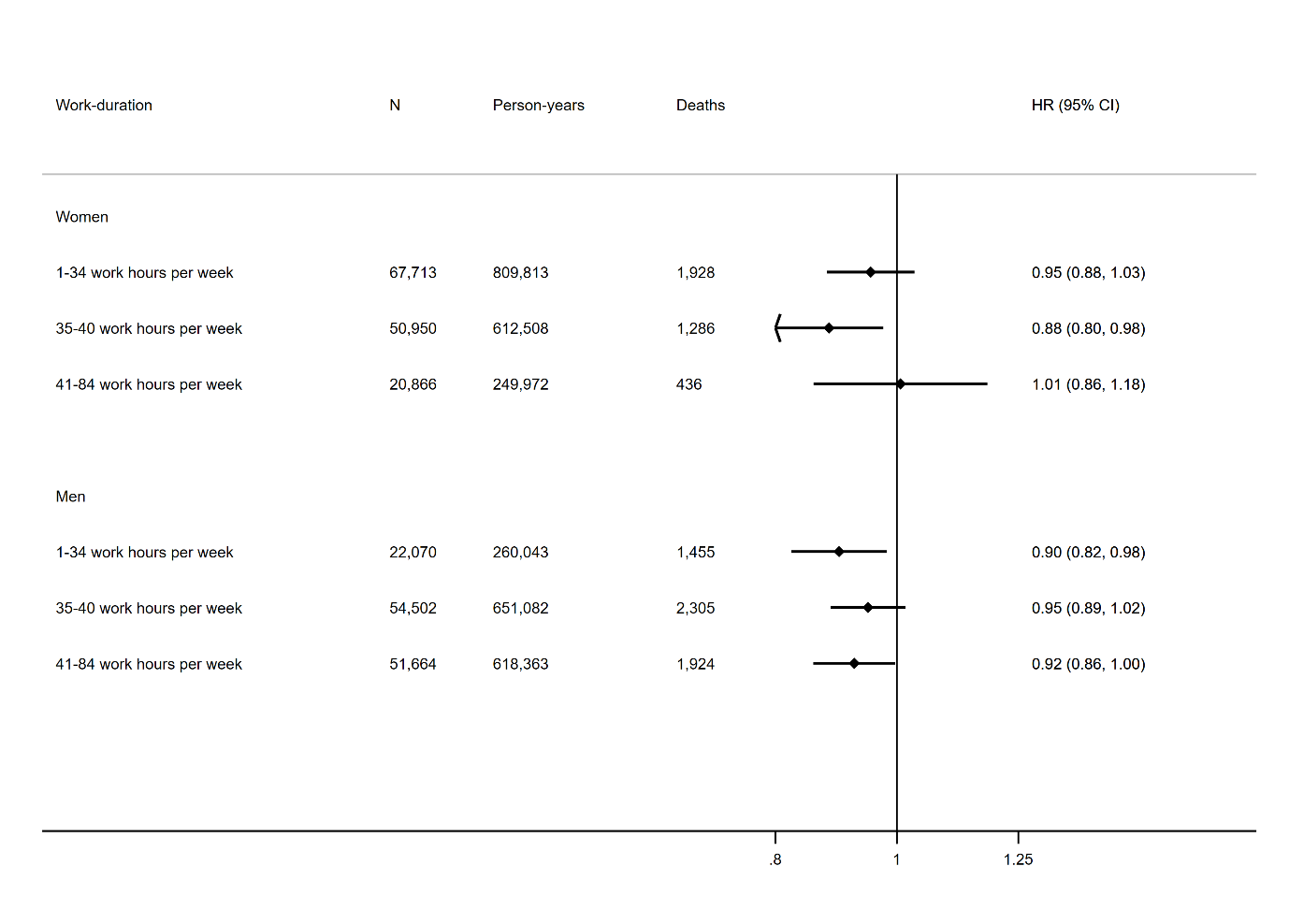 |
| --- |
| **Figure S15** Hazard ratio (HR) and 95% confidence interval (CI) of all-cause mortality per 5 kJ/day/kg of non-occupational physical activity energy expenditure across tertiles of working hours per week. Model 2 hazard ratios are adjusted for age (underlying timescale), ethnicity, Townsend deprivation index, highest educational level (stratified baseline hazard), annual household income (stratified baseline hazard), years in current job, job involves shift work, alcohol consumption, smoking, salt added to food, oily fish intake, fruit and vegetable intake (stratified baseline hazard), processed and red meat intake, parental history of cancer or cardiovascular disease, use of blood pressure or cholesterol lowering medications, doctor-diagnosed diabetes or treatment with insulin, baseline prevalent cancer, baseline prevalent cardiovascular disease, occupational physical activity category, body mass index, resting heart rate. Arrow indicates confidence interval boundary out of range. |

| 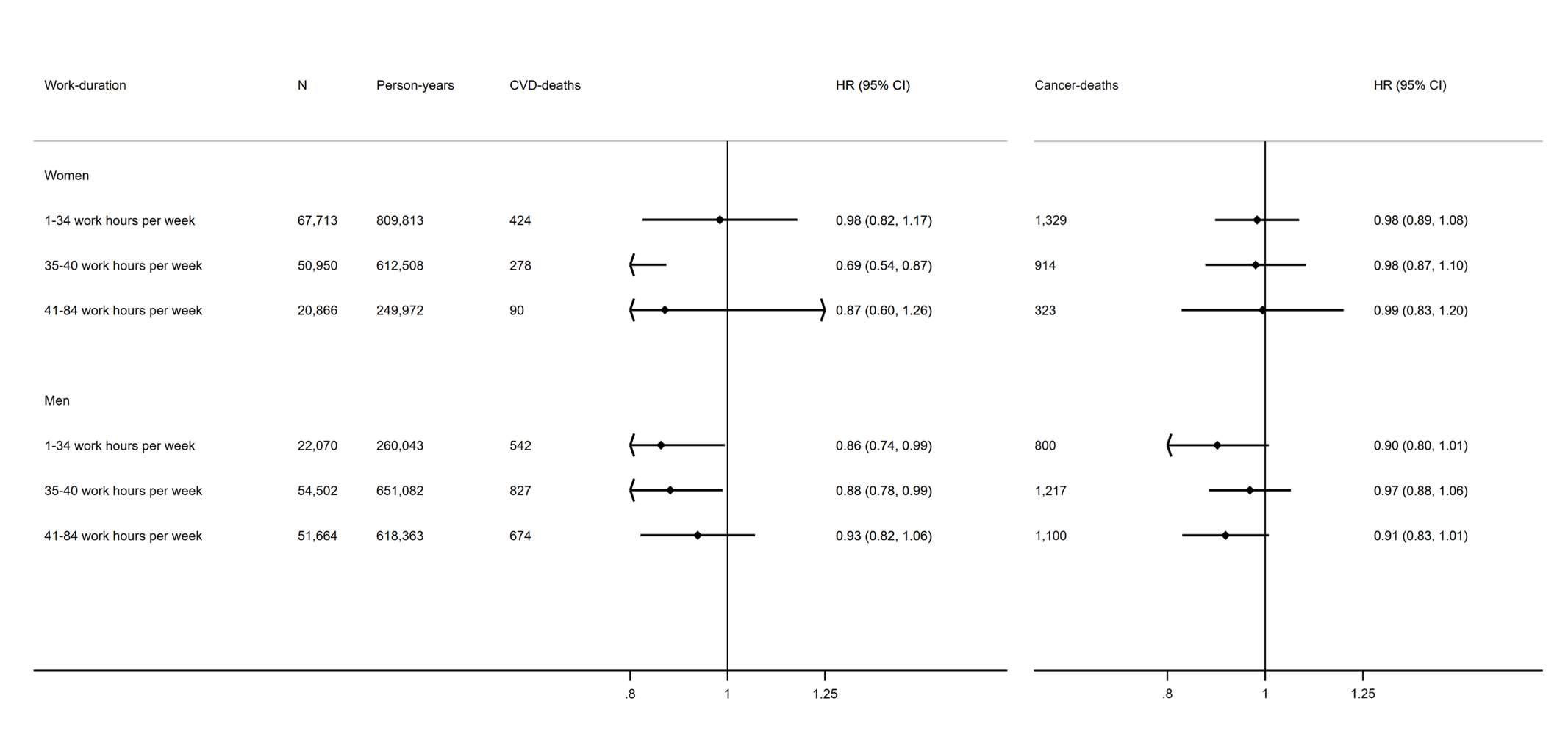 |
| --- |
| **Figure S16** Hazard ratio (HR) and 95% confidence interval (CI) of cardiovascular disease mortality (left) and cancer mortality (right) per 5 kJ/day/kg of non-occupational physical activity energy expenditure across tertiles of work duration in hours per week. Model 2 hazard ratios are adjusted for age (underlying timescale), ethnicity, Townsend deprivation index, highest educational level (stratified baseline hazard), annual household income (stratified baseline hazard), years in current job, job involves shift work, alcohol consumption, smoking, salt added to food, oily fish intake, fruit and vegetable intake (stratified baseline hazard), processed and red meat intake, parental history of cancer or cardiovascular disease, use of blood pressure or cholesterol lowering medications, doctor-diagnosed diabetes or treatment with insulin, baseline prevalent cancer, baseline prevalent cardiovascular disease, occupational physical activity category, body mass index, resting heart rate. Arrow indicates confidence interval boundary out of range. Interaction of non-occupational physical activity energy expenditure by tertile of work duration: cardiovascular disease in women in (p=0.92), cancer in women (0.75), cardiovascular disease in men (p=0.03), cancer in men (0.55). |

**Supplemental references**

1. Pearce M, Strain T, Kim Y, Sharp SJ, Westgate K, Wijndaele K, et al. Estimating physical activity from self-reported behaviours in large-scale population studies using network harmonisation: findings from UK Biobank and associations with disease outcomes. Int J Behav Nutr Phys Act. 2020;17(40).
2. White T, Westgate K, Wareham NJ, Brage S. Estimation of physical activity energy expenditure during free-living from wrist accelerometry in UK adults. PLoS One. 2016;11(12):e0167472
3. White T, Westgate K, Hollidge S, Venables M, Olivier P, Wareham N, et al. Estimating energy expenditure from wrist and thigh accelerometry in free-living adults: a doubly labelled water study. Int J Obes. 2019. 43, pages2333–2342
4. Doherty A, Jackson D, Hammerla N, Plötz T, Olivier P, Granat MH, et al. Large scale population assessment of physical activity using wrist worn accelerometers: the UK Biobank Study. PLoS One. Public Library of Science; 2017 Feb 1;12(2):e0169649.
5. van Hees VT, Fang Z, Langford J, Assah F, Mohammad A, da Silva ICM, et al. Autocalibration of accelerometer data for free-living physical activity assessment using local gravity and temperature: an evaluation on four continents. J Appl Physiol [Internet]. American Physiological Society; 2014;117(7):738–44.
6. van Hees VT, Gorzelniak L, Dean Leon EC, Eder M, Pias M, Taherian S, et al. Separating movement and gravity components in an acceleration signal and implications for the assessment of human daily physical activity. PLoS One. 2013. 2013;8(4):e61691.
7. Brage S, Westgate K, Wijndaele K, Godinho J, Griffin S WN. Evaluation of a method for minimising diurnal information bias in objective sensor data. In: ICAMPAM. Amherst; 2013.
